# Regulation of beige adipocyte thermogenesis by the cold-repressed ER protein NNAT

**DOI:** 10.1016/j.molmet.2023.101679

**Published:** 2023-01-25

**Authors:** Kyung-Mi Choi, Christopher Y. Ko, Sung-Min An, Seung-Hee Cho, Douglas J. Rowland, Jung Hak Kim, Anna Fasoli, Abhijit J. Chaudhari, Donald M. Bers, John C. Yoon

**Affiliations:** 1 Division of Endocrinology, Department of Internal Medicine, University of California Davis School of Medicine, Davis, CA 95616, USA; 2 Institute of Molecular Biology and Genetics, School of Biological Sciences, Seoul National University, Seoul 08826, South Korea; 3 Department of Pharmacology, University of California Davis School of Medicine, Davis, CA 95616, USA; 4 Center for Molecular and Genomic Imaging, Department of Biomedical Engineering, University of California Davis, Davis, CA 95616, USA; 5 Department of Radiology, University of California Davis School of Medicine, Sacramento, CA 95825, USA

**Keywords:** NNAT, SERCA2, NHLRC1, Thermogenesis, Beige fat

## Abstract

**Objective:**

Cold stimuli trigger the conversion of white adipose tissue into beige adipose tissue, which is capable of non-shivering thermogenesis. However, what process drives this activation of thermogenesis in beige fat is not well understood. Here, we examine the ER protein NNAT as a regulator of thermogenesis in adipose tissue.

**Methods:**

We investigated the regulation of adipose tissue NNAT expression in response to changes in ambient temperature. We also evaluated the functional role of NNAT in thermogenic regulation using *Nnat* null mice and primary adipocytes that lack or overexpress NNAT.

**Results:**

Cold exposure or treatment with a β3-adrenergic agonist reduces the expression of adipose tissue NNAT in mice. Genetic disruption of *Nnat* in mice enhances inguinal adipose tissue thermogenesis. *Nnat* null mice exhibit improved cold tolerance both in the presence and absence of UCP1. Gain-of-function studies indicate that ectopic expression of *Nnat* abolishes adrenergic receptor-mediated respiration in beige adipocytes. NNAT physically interacts with the ER Ca^2+^-ATPase (SERCA) in adipocytes and inhibits its activity, impairing Ca^2+^ transport and heat dissipation. We further demonstrate that NHLRC1, an E3 ubiquitin protein ligase implicated in proteasomal degradation of NNAT, is induced by cold exposure or β3-adrenergic stimulation, thus providing regulatory control at the protein level. This serves to link cold stimuli to NNAT degradation in adipose tissue, which in turn leads to enhanced SERCA activity.

**Conclusions:**

Our study implicates NNAT in the regulation of adipocyte thermogenesis.

## Highlights

• The adipose expression of NNAT is suppressed by cold and β-adrenergic signaling.
• Mice lacking NNAT exhibit greater inguinal adipose tissue thermogenesis.
• NNAT inhibits ER calcium transport and thermogenic respiration in beige adipocytes.
• NHLRC1 is induced by cold and regulates NNAT levels.

## 1 Introduction

Increasing energy expenditure has emerged as a promising therapeutic strategy against excess weight gain and associated metabolic abnormalities [1]. It is understood that white adipocytes store energy as triglycerides whereas brown adipocytes dissipate energy in the form of heat, a process known as adaptive thermogenesis [2]. Certain conditions, such as cold exposure or treatment with β-adrenergic agonists, can stimulate the formation of beige adipocytes within white adipose tissue [WAT) depots, which then display the capacity for adaptive thermogenesis [3]. Adult humans possess beige-like adipocytes which can be recruited by various stimuli including chronic cold exposure, bariatric surgery, cancer cachexia, and burn-induced cachexia [4]. Therefore, promoting the beiging of white adipocytes represents a potentially effective approach to managing and preventing obesity and metabolic disorders such as type 2 diabetes.

Over the past several decades, studies of uncoupling protein 1 (UCP1), an inner mitochondrial membrane protein that dissipates the proton gradient upon activation, have provided insights into the mechanisms of thermogenic function in adipocytes [5]. UCP1 is present in brown and beige adipocytes but not in white adipocytes [6]. More recent data indicate that UCP1, while an important contributor, is dispensable for adipocyte thermogenesis, and point to several UCP1-independent mechanisms [[7], [8], [9]]. In particular, futile creatine cycling and sarco/endoplasmic reticulum Ca^2+^-ATPase (SERCA)-mediated calcium cycling have been implicated in beige adipocyte thermogenesis [8, 9].

Because of the inducible nature of beige adipocyte development and the known presence of beige-like adipocytes in adult humans, it may be possible to target beige adipocyte-selective regulatory pathways to achieve medical benefits. A number of genes that promote browning have been identified in recent years, notably *Prdm16* [10], but carrying out gene therapy in adipose tissue is challenging. We have been interested in finding genes that suppress thermogenesis because inhibitors of such proteins may have therapeutic potential. One such gene is *Nnat*, which encodes a small endoplasmic reticulum (ER) membrane protein and is the most strongly regulated among the genes downregulated by cold exposure in our transcriptome analysis of inguinal adipose tissues. NNAT is highly expressed in WAT, at lower levels in the brain and pancreatic β-cells, and little elsewhere including brown adipose tissue (BAT) [11, 12]. Also known as neuronatin, NNAT has been implicated in central nervous system (CNS) development and neural differentiation of embryonic stem cells [13]. NNAT has significant amino acid sequence homology to phospholamban and sarcolipin, both small sarcoplasmic reticulum (SR) membrane proteins that bind SERCA and modulate calcium transport in the heart and skeletal muscle, respectively [[14], [15], [16]]. NNAT has also been suggested to be involved in muscle thermogenesis [17,18]. We hypothesized that NNAT in WAT may control adaptive thermogenesis in adipose tissue by interacting with SERCA to regulate calcium cycling in the ER. Whereas SERCA is expressed widely [19], being especially abundant in cardiac tissues, NNAT shows tissue-selective expression, making it an attractive therapeutic target.

Here we demonstrate that loss of NNAT enhances the thermogenic capacity of beige adipocytes in culture and in mice. The NNAT expression level in adipose tissue is suppressed by lowering the ambient temperature or administration of β-adrenergic agonists. Ectopic expression of *Nnat* in cultured beige adipocytes reduces β-adrenergic receptor-stimulated cellular respiration, which we attribute to the binding of NNAT to SERCA to inhibit calcium flux into the ER. In the absence of NNAT, ER calcium flux and oxygen consumption are enhanced. We also report that cold exposure induces NHLRC1, an E3 ubiquitin ligase implicated in proteasomal degradation of NNAT. Together, these data delineate a pathway by which NNAT controls thermogenesis in adipocytes, thereby suggesting a potential new target in treating and preventing obesity.

## 2 Materials and methods

### 2.1 Mice and diets

All animal experiments were performed according to the protocols approved by the UC Davis Institutional Animal Care and Use Committee (IACUC). The global *Nnat* knockout mice were generated by the UC Davis Mouse Biology Program. The *Ucp1* knockout mice were obtained from the Jackson Laboratory (B6.129-*Ucp1*^*tm1Kz*^/J, stock 003124). Mice were maintained on a standard chow (Teklad Global Rodent Diets) at the indicated temperature under a 12 h light/12 h dark cycle and used between 8 and 12 weeks of age unless otherwise noted. Littermate controls were used in all cases.

### 2.2 Primary cell culture

Primary stromal vascular fractions (SVF) from iWAT of 3 to 4-week-old *Nnat* KO or WT mice were isolated and cultured following a beige adipocyte differentiation protocol [20]. Briefly, SVF cells were differentiated by treating fully confluent cells with differentiation induction medium (DMEM containing 10% fetal bovine serum (FBS), 5 μg/ml insulin, 0.5 mM isobutylmethylxanthine (IBMX), 5 μM dexamethasone, 125 μM indomethacin, 1 nM 3,3′,5-triiodo-l-thyronine (T3) and 1 μM rosiglitazone) at day 0. After 2 days, the medium was changed to differentiation maintenance medium (DMEM containing 10% FBS, 5 μg/ml insulin, 1 nM T3 and 1 μM rosiglitazone). The medium was replenished every two days.

### 2.3 Lentivirus production

To generate a lentiviral construct expressing mouse *Nnat*, we isolated iWAT from three 9-week-old C57BL/6J male mice housed at 30 °C for 1 week. We synthesized the *Nnat* cDNA by reverse transcription of pooled mRNA from the iWAT using SuperScript IV reverse transcriptase (Invitrogen). The *Nnat* cDNA was amplified by PCR and cloned into the PCR8 entry vector using a TA cloning kit (Invitrogen). Then, we generated mammalian cell expression plasmid encoding *Nnat* by performing LR recombination reaction between the entry vector and the destination vector pLenti-CMV-Puro-DEST (Addgene #17452) using Gateway LR clonase enzyme mix (Invitrogen). To produce the *Nnat*-expressing lentivirus and the *LacZ* control virus, we transfected 293FT cells with a packaging construct (psPAX2; Addgene #12260), an envelope protein-producing plasmid (pCMV-VSV-G; Addgene #8454) and either pLenti-CMV-*Nnat*-Puro or pLenti-CMV-*LacZ*-Puro plasmid, respectively. We collected culture medium containing lentivirus at 48 h and 72 h after initial transfection. After centrifugation, the viral supernatant was passed through a 45 μm filter and stored at −80 °C until use. For silencing of *Nhlrc1*, we used a pLKO.1 puro vector (Addgene #8453) containing an shRNA cassette that targets mouse *Nhlrc1* (5′-CCGCAGTGATAATTTGAGAAA-3′) and a scrambled control shRNA vector (Addgene #1864), and produced the lentivirus using the same procedures.

### 2.4 Transduction of primary adipocytes with lentiviruses

For transient expression of the *Nnat* cDNA or an *Nhlrc1-*targeting shRNA in differentiated primary iWAT cells, the viral supernatant was diluted 3-fold with DMEM containing 10% FBS, 8 μg/ml polybrene, 5 μg/ml insulin, 1 nM T3 and 1 μM rosiglitazone and added to the cells twice at day 3 and day 4. On the next day of final transduction (day 5), cells were replenished with fresh DMEM containing 10% FBS, 5 μg/ml insulin, 1 nM T3 and 1 μM rosiglitazone. We changed the medium every 2 days until cells were fully differentiated.

### 2.5 Cell transfection

293FT cells (Invitrogen, R70007) were grown to 90% confluence and were co-transfected with 2 μg of pLenti-CMV-*Nnat*-Puro and various amounts (0–2 μg) of pLenti-CMV-*Nhlrc1*-Flag-Puro vectors. The total DNA used for transfection was adjusted equally to 4 μg by adding pLenti-CMV-*LacZ*-Puro vector. Cells were transfected with TransIT 293 transfection reagent (Mirus) and were harvested 48 h after transfection.

For INS-1 cell transfection, cells were cultured in RPMI-1640 medium supplemented with 10% FBS until 70% confluence. Cells were then transfected with pcDNA3.1-*Nhlrc1*-Flag vector (GenScript) using Lipofectamine 2000 reagent. The empty pcDNA3.1-Flag vector (GenScript) was used for equal loading of total DNA (4 μg) into the cells. The transfection medium was changed with normal medium after 5 h, the cells were harvested at 48 h post-transfection for immunoblotting.

### 2.6 Oxygen consumption measurements

The oxygen consumption rate (OCR) was measured using a respirometer (Strathkelvin Instruments, MT200) equipped with a Clark-type electrode (Strathkelvin Instruments, SI130) in differentiated primary iWAT cells in the absence or presence of indicated chemicals. Cells were trypsinized, centrifuged, and resuspended in 1 ml of respiration medium consisting of high glucose DMEM (Gibco, 10569044) and 2% bovine serum albumin. 1 × 10^6^ viable cells in 0.6 ml of respiration medium were transferred to a magnetically stirred respiration chamber equilibrated to 37 °C and the oxygen concentration in the chamber was monitored for 5 min. Simultaneous recordings were made with two electrodes and measurements from at least 6 runs were combined for analysis. Data were analyzed by Strathkelvin 782 system data analysis module (version 4.1). The OCR values were obtained by calculating the rate of decrease in the oxygen concentration and compared between conditions.

### 2.7 Cytosolic calcium measurements

Fully differentiated iWAT cells with the indicated genotype were seeded at 40,000 cells per well in collagen I-coated 96-well black clear-bottom plates and incubated overnight. Intracellular calcium concentration ([Ca]_i_) was monitored with the [Ca]_i_ indicator Fluo-4 AM (F14201, ThermoFisher). Cells were loaded with 5 μM Fluo-4 AM and 0.02% Pluronic F-127 (P3000MP, ThermoFisher) for 30 min at 37 °C in Hank's balanced salt solution (HBSS). Cells were then washed twice and incubated in HBSS for 30 min at room temperature. Before fluorescence recording, the buffer was changed with calcium-free HBSS. Following baseline recording (F_0_), 2 μM thapsigargin or 50 μM 2-APB was added at the indicated time-point and then the fluorescence was recorded. Fluorescence signals were measured with a Spectramax M5 multimode plate reader (Molecular Devices; excitation, 494 nm; emission, 516 nm).

### 2.8 Caged IP_3_ photoactivation or phospholipase C activator treatment

Hela cells expressing *Nnat* or *LacZ* were seeded at 30,000 cells per well in collagen I-coated 96-well plates and incubated overnight. Cells were loaded with 3 μM ci-IP_3_/PM (Tocris Bioscience) and 0.02% Pluronic F-127 for 45 min at room temperature. 5 μM Fluo-4 AM and 0.02% Pluronic F-127 were then added to cells and incubated for a further 45 min at room temperature. After washing and de-esterification for 30 min, baseline fluorescence (F_0_) was recorded with an M5 multimode plate reader. The caged IP_3_ was uncaged by UV exposure (365 nm) for 15 s and then the fluorescence was recorded for 3 min. For phospholipase C activator treatment, HeLa cells on the 96-well plate were prepared as above. Cytosolic calcium was measured as above. The fluorescence signal was recorded before and after treatment of 25 μM m-3M3FBS (Sigma).

### 2.9 Chronic and acute cold challenge

For chronic cold exposure, 10-week-old male mice previously housed at 22 °C were group-housed at 7 °C for 3 weeks with free access to food and water. For an acute cold challenge, male mice at 5 weeks of age were acclimated at 30 °C for 3 weeks and then singly placed at 7 °C for 24 h or 48 h with free access to food and water. Tissues were then harvested for RNA and protein analysis.

### 2.10 Treatment with the β3-adrenergic agonist CL316,243

CL316,243 or saline was injected intraperitoneally to age-matched male mice at a dose of 1 mg/kg body weight for 3 or 7 consecutive days. On the day after the last injection, the mice were sacrificed.

### 2.11 Core body temperature

For assessment of adaptation to mild cold, 10-week-old *Nnat* KO and WT mice were acclimated at 30 °C for 4 weeks before acute cold exposure. On the day of the cold tolerance test, mice were singly placed at 22 °C without food and with free access to water. Rectal core body temperature was monitored using a thermometer (Bioseb) every hour for 5 h. For assessment of adaptation to severe cold, we transferred mice kept at 22 °C to 7 °C in pre-chilled cages with free access to water only. Recording of the core temperature was performed in the same manner.

### 2.12 Measurement of tissue temperature

To measure the local tissue temperature directly, we implanted thermocouple probes (Sable systems international, TC-2000) in BAT and iWAT of mice anesthetized with isoflurane. Temperature changes following intraperitoneal injection of CL316,243 (1 mg/kg body weight) were recorded in real-time.

### 2.13 Indirect calorimetry

O_2_ consumption, CO_2_ production, food intake, and physical activity were evaluated in the male *Nnat* KO and WT mice fed chow diet by indirect respiratory calorimetry in the Comprehensive Lab Animal Monitoring System (CLAMS, Columbus Instruments). O_2_ consumption and CO_2_ production were measured and used to calculate respiratory exchange ratio and energy expenditure [21]. Mice were acclimated to the facility for at least 1 week at 30 °C prior to initiation of calorimetry at 11 weeks of age. Measurements were taken for 48 h (2 light/2 dark cycles) at three environmental temperatures (30, 22, and 10 °C). The body composition was assessed by dual-energy X-ray absorptiometry (DEXA) under isoflurane anesthesia, using a Lunar PIXImus II Densitometer (GE Medical Systems) immediately after completion of the indirect calorimetry measurements.

### 2.14 ^18^F-FDG-PET imaging and analysis

Mice were fasted overnight for approximately 12 h and were injected with CL316,243 (1 mg/kg body weight) at the start of the fast and again the following morning, 1 h before the positron emission tomography (PET) study. Animals were then injected with 0.3 mCi of [^18^F]Fluorodeoxy-glucose and a 15 min static PET image was obtained 45 min later. Animals were anesthetized with 1.5–2.0% isoflurane and maintained with 1.0–1.5% isoflurane for the duration of imaging. WT and KO mice were imaged side-by-side in one of two preclinical PET systems (Inveon Dedicated PET from Siemens or microPET Focus 120 from CTI-Concorde Microsystems LLC). PET data were reconstructed using 2 iterations of a 3-D ordered subset expectation maximization (OSEM3D) followed by 18 iterations of a maximum a posteriori (MAP) algorithm. The imaging matrix was 128 × 128 × 95 with reconstructed voxel sizes of 0.43 mm × 0.43 mm × 0.80 mm.

Image processing and analysis were performed using PMOD v.4.102 (PMOD Technologies). Individual animals were digitally separated. A 2 mm spherical volume-of-interest (VOI) was placed over the central hot spot of the left and right iWAT. The standardized uptake value (SUV) of the 8 hottest voxels in this 2 mm sphere was determined for each iWAT. Prism 9 (GraphPad Software, LLC) was used to perform a Mann–Whitney two-tailed analysis comparing WT to KO animals. The images shown are from two representative individuals with the SUV value closest to the group mean; their cross sections do not align exactly. The bladder uptake is due to FDG excretion and is expected.

### 2.15 Quantitative real-time PCR

Total RNA was isolated using Trizol (Invitrogen) followed by removal of genomic DNA using gDNA eliminator spin column (Qiagen). The cDNA was synthesized using SuperScript IV reverse transcriptase (Invitrogen). qPCR was performed using SYBR Green (Bio-Rad) in a Bio-Rad CFX real-time PCR system. Primers used in this study are listed in Supplementary Table 1.

### 2.16 TaqMan microRNA assay

For small RNA assays, we extracted RNA from iWAT of cold-exposed mice using miRNeasy mini kit (Qiagen). Reverse transcription was conducted by using TaqMan MicroRNA reverse transcription kit (ThermoFisher) and RT primers (*miR-708-5p*, assay ID 002341; *snoRNA202* as an internal control, assay ID 001232) included in TaqMan MicroRNA assay (ThermoFisher). Quantitative PCR reaction was set by using TaqMan Universal Master mix without UNG (ThermoFisher) and the fluorescence signals were read in a Bio-Rad CFX real-time PCR machine.

### 2.17 RNA-sequencing and data processing

Total RNA samples from iWAT of mice housed at 30 °C and exposed to chronic cold were isolated using Trizol (Invitrogen) and purified with DNase-treated spin column (Zymo Research). Sequencing libraries were prepared and high-throughput 3′-Tag-sequencing was performed using a HiSeq 4000 system (Illumina).

The raw single-end reads were quality-checked using fastqc and trimmed to remove adapter sequences. The processed reads were aligned to GRCm38 primary genome assembly using GENCODE v21 annotation to generate counts per gene. Differential expression analysis was conducted using the limma-voom Bioconductor pipeline [22]. The volcano plot was created using R (version 3.4.3). The RNA-seq data were deposited to Gene Expression Omnibus under the accession number of GSE133619.

### 2.18 Protein extraction and western blotting

Mouse tissues were homogenized in 400 μl of RIPA buffer (Sigma) supplemented with 1 mM phenylmethylsulfonyl fluoride (Sigma), protease inhibitor (Roche) and PhosSTOP cocktail (Roche), followed by sonication and centrifugation. For cell lysates, we added 30–100 μl of the same lysis buffer to cell pellet depending on the pellet size and incubated on ice for 10 min. After centrifugation, the supernatant was collected and quantitated by Pierce BCA protein assay kit (Life Technologies). Ten micrograms of total protein were loaded on SDS-PAGE gel unless otherwise noted. We used NNAT monoclonal antibody (Abcam, 1:3000 dilution), NHLRC1 polyclonal antibody (Fisher Scientific, 1:1000), NHLRC1 monoclonal antibody (Fisher Scientific, 1:1000), SERCA2 monoclonal antibody (Cell Signaling, 1:1000), RYR1 monoclonal antibody (Fisher Scientific, 1:1000), RYR2 monoclonal antibody (Sigma, 1:1000), IP3R1 monoclonal antibody (Cell Signaling, 1:1000), UCP1 monoclonal antibody (R&D systems, 1:3000), FABP4 monoclonal antibody (Santa Cruz, 1:3000), and β-actin monoclonal antibody (Santa Cruz, 1:1000). Goat anti-mouse IgG (Jackson ImmuoResearch) and goat anti-rabbit IgG (Jackson ImmunoResearch) were used as secondary antibodies. Immunoblots were developed with Luminata Crescendo Western HRP substrate (Millipore Sigma) or SuperSignal West Femto Maximum Sensitivity Substrate kit (Fisher Scientific). The protein signals were detected using a Bio-Rad ChemiDoc system.

### 2.19 Immunoprecipitation

The iWAT lysate was prepared by homogenization in immunoprecipitation (IP) lysis buffer (50 mM HEPES pH 7.4, 150 mM NaCl, 10% glycerol, 1.5 mM MgCl_2_, 1 mM EDTA, 1 mM EGTA, 1% Triton X-100, 100 mM sodium fluoride, 1 mM sodium orthovanadate, 1 × protease inhibitor and 1 × phosphatase inhibitor). The lysate was immunoprecipitated using Dynabeads protein A (ThermoFisher) with SERCA2 polyclonal antibody (Abcam) or rabbit IgG antibody (Cell Signaling). The resulting IP samples were loaded to SDS-PAGE gel and the immunoblot was probed with the NNAT monoclonal antibody (Abcam) and SERCA2 monoclonal antibody (Cell Signaling). For detection of interaction between NNAT and NHLRC1, differentiated iWAT cells were treated with 10 μM MG132 for 4 h before cell lysis. During the IP process, Dynabeads and NNAT polyclonal antibody (Abcam) were crosslinked using BS3 crosslinker (ThermoFisher). NHLRC1 polyclonal antibody and NNAT monoclonal antibody were used for immunoblotting.

### 2.20 Chemical crosslinking

Crosslinking experiments were performed as previously described [23]. iWAT was collected from 3-month-old C57BL/6J female mice, rinsed with cold PBS and homogenized in PBS buffer containing 145 mM NaCl, 10% glycerol, 1 mM MgCl_2_, 5 mM EDTA, 1 mM PMSF and protease/phosphatase inhibitors. Crosslinker BMH (Fisher Scientific) was added at a final concentration of 0.4 mM into 1.75 mg tissue lysate, incubated at room temperature for 1 h with rotation and stored at 4 °C overnight. On the next day, 2% Triton X-100 was added to the cross-linked lysate and proceeded to immunoprecipitation as described above.

### 2.21 Cycloheximide chase assay

Differentiated iWAT cells were treated with DMSO or 10 μM MG132 (Cell Signaling) for 4 h before the addition of 100 μg/mL cycloheximide (Calbiochem) and 10 μM CL316,243. Cells were harvested and lysed for protein extraction.

### 2.22 Ubiquitination assay

293FT cells were transfected with 2 μg each of HA-Ubiquitin and pLenti-CMV-Nnat-Puro, along with pcDNA3.1-Nhlrc1-Flag or empty pcDNA3.1-Flag vector using TransIT 293 transfection reagent (Mirus). Cells were incubated for 48 h, followed by the treatment with 10 μM MG132 for 6 h before harvest. Cell lysate (500 μg) was used for IP with a polyclonal NNAT antibody and ubiquitinated NNAT was detected by immunoblot analysis with a monoclonal HA antibody (Cell Signaling, 1:1000). IP buffer for ubiquitination assay included 10 mM NEM (Sigma).

### 2.23 Statistical analysis

Data were presented as mean ± SD, except for line graphs, which were presented as mean ± SEM. Two-tailed Student's *t*-test was performed to analyze the difference between two independent groups unless otherwise noted. Mann–Whitney two-tailed analysis was applied for PET data analysis. For CLAMS data, *p*-value was determined by ANCOVA analysis with lean mass as a covariate. Two-way ANOVA followed by Fisher's LSD test was used for calcium level comparisons. ∗*P* ≤ 0.05, ∗∗*P* ≤ 0.01, ∗∗∗*P* ≤ 0.001. Statistical significance was considered at *P* ≤ 0.05.

## 3 Results

### 3.1 NNAT expression in adipose tissue is strongly repressed by cold stress and β3-adrenergic stimulation

**Figure 1:**
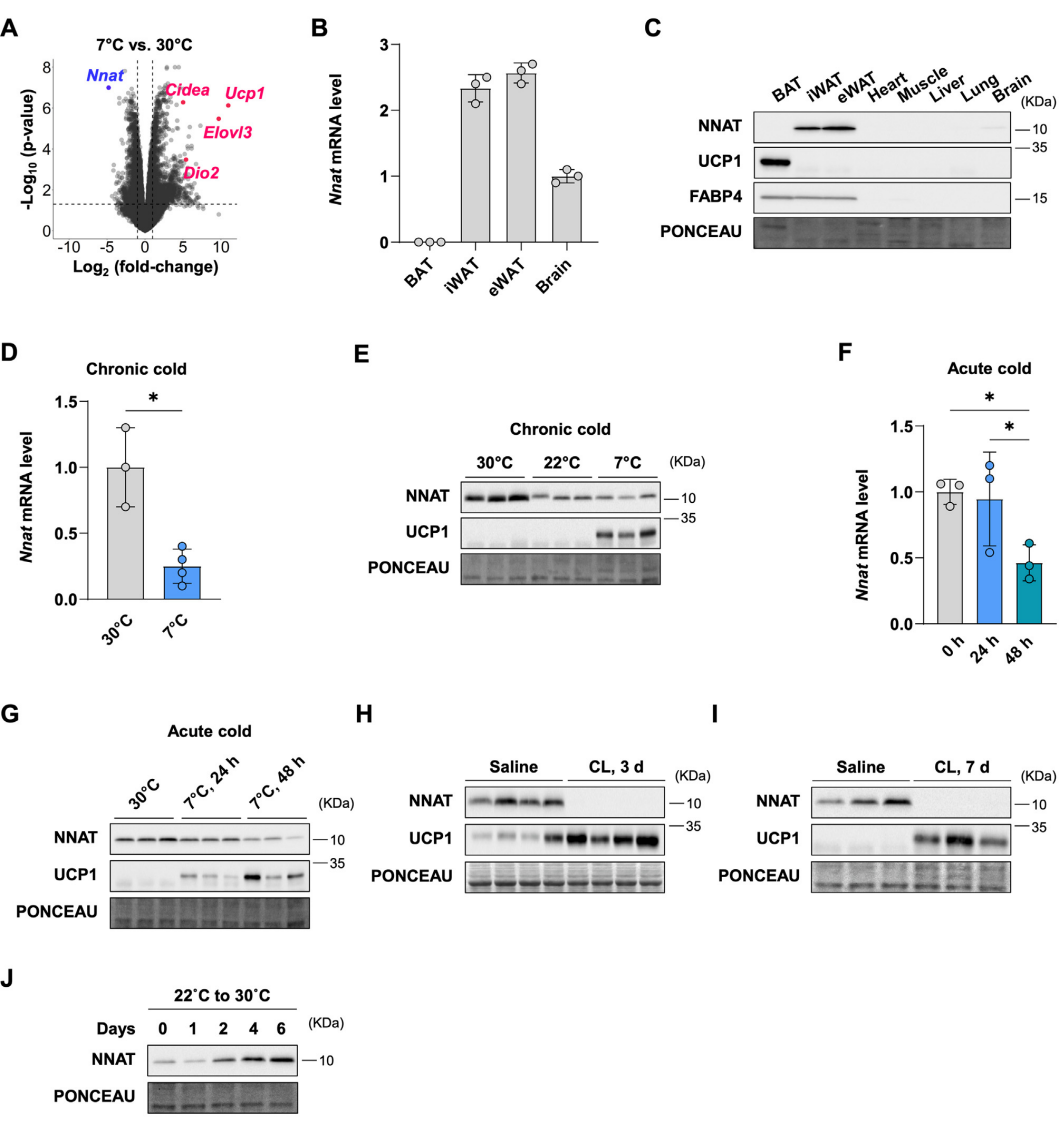
**NNAT expression is highly enriched in white fat and repressed during cold exposure (A) Volcano plot of RNA-seq data**. Nine-week-old male mice were maintained at thermoneutrality (30 °C) or acclimated to cold (7 °C) for 3 weeks (n = 3 for both groups). The log_2_ fold-changes at 7 °C compared to at 30 °C are indicated on the x-axis. The y-axis represents the log_10_ p-values. The dotted lines on the x- and y-axes show a 2-fold change and p-value of 0.05, respectively. (B) Relative mRNA levels of *Nnat* in the indicated tissues. (C) NNAT protein levels in various tissues. (D) The mRNA levels of *Nnat* in iWAT exposed to chronic cold or housed at 30 °C. (E) Expression of NNAT in iWAT determined by immunoblotting under 30 °C, 22 °C, or 7 °C conditions for 3 weeks. (F and G) The expression of *Nnat* in iWAT after acute cold exposure determined by qPCR (F) and immunoblotting (G). (H and I) NNAT protein levels in iWAT of mice administered to saline or CL316,243 for 3 days (H) and 7 days (I). (J) NNAT protein levels in iWAT after acclimation from 22 °C to 30 °C for 0–6 days. *P*-values are determined by two-tailed Student's *t*-test.

We sought to identify proteins that suppress thermogenesis, reasoning that inhibitors of such proteins may have therapeutic utility. To find possible candidates for negative regulators of thermogenesis, we performed RNA-sequencing on inguinal white adipose tissue (iWAT) from mice exposed to cold for a prolonged period (7 °C for 3 weeks) compared to controls housed at thermoneutrality (30 °C). We observed 1,391 upregulated genes and 1,150 downregulated genes in chronically cold-exposed iWAT. A marked induction of classic thermogenic genes including *Ucp1* and *Elovl3* (Figure 1A) was seen, confirming beiging of white adipose tissue. Notably, the most strongly suppressed gene was *Nnat*, which decreased 28-fold with cold exposure (Figure 1A). *Nnat* is expressed in iWAT, epididymal WAT (eWAT), and to a lesser extent, the brain of mice raised at room temperature (22 °C) (Figure 1B,C). Its expression in BAT is low at both mRNA and protein levels (Figure 1B,C).

We validated the changes in the *Nnat* expression in iWAT following chronic and acute cold exposure by qPCR and immunoblotting. Compared to 30 °C, the *Nnat* mRNA and protein levels were suppressed following chronic cold exposure (Figure 1D,E). They also declined within 48 h upon acute cold exposure (Figure 1F,G), indicating that the regulation of the NNAT expression *in vivo* occurs relatively rapidly under cold stress. In addition, the iWAT NNAT protein level *in vivo* was suppressed by short-term and long-term treatment with CL316,243, a β3-adrenergic agonist that elicits many of the physiological responses to cold exposure in adipose tissue, including lipolysis and thermogenesis (Figure 1H,I). Next, we investigated the effect of warming on the NNAT protein expression in iWAT by moving the mice from 22 °C to 30 °C and observed a significant induction of the NNAT level by day 2 (Figure 1J). We also assessed the NNAT levels in BAT and hypothalamus in response to cold stimuli. The *Nnat* mRNA level in BAT declined with both chronic and acute cold exposure (Supplementary Figs. 1A and B). At the protein level, NNAT is readily detected at 30 °C, at which BAT is thermogenically inactive, and difficult to detect at 22 °C and 7 °C (Supplementary Figs. 1C and D). The NNAT expression in hypothalamus was unaffected by cold (Supplementary Figure 1E). Collectively, our observations suggest that the *Nnat* expression in adipose tissue is highly sensitive to ambient temperature, correlating negatively with thermogenesis *in vivo*.

### 3.2 Loss of NNAT enhances thermogenesis in adipose tissue

**Figure 2:**
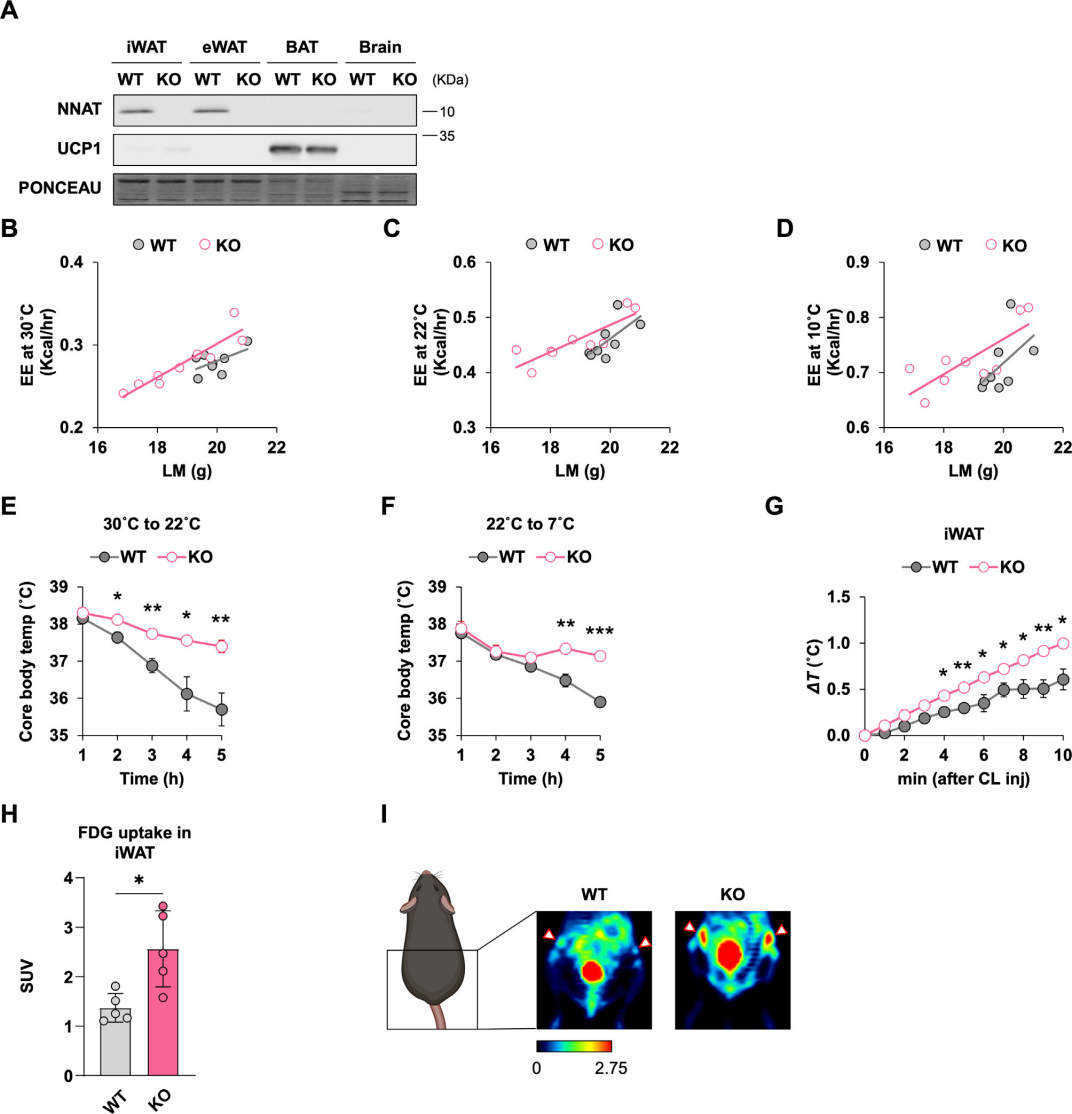
**Loss of NNAT enhances thermogenic function *in vivo*** (A) NNAT protein level in indicated tissues of *Nnat* KO mice and WT-littermate. (B to D) Metabolic cage studies of *Nnat* KO (n = 9) and WT-littermate (n = 8). Raw energy expenditure (EE) data were plotted relative to lean mass (LM) and fitted by linear regression (B, C, D). ANCOVA analysis with lean mass as a covariate was utilized to calculate *P*-values. (E and F) Effect of acute cold exposure from 30 °C to 22 °C (G) and that from 22 °C to 7 °C (H) on core rectal temperature of *Nnat* KO and WT-littermate (n = 5 for both groups). (G) Changes in the iWAT temperature following CL316,243 treatment. WT-littermate, n = 3; *Nnat* KO, n = 4. (H) FDG-PET standardized uptake value (SUV) measures for iWAT from *Nnat* KO (n = 5) and WT-littermate (n = 5) groups. The Mann–Whitney test reveals a significant increase in FDG uptake for KO compared to WT (*p* = 0.016). (I) Coronal FDG-PET images of the chest and abdomen for representative animals from the two groups in (E). From each group, the individual with the SUV value closest to the group mean is shown. Due to differences in animal positioning, the cross section images for the two animals do not necessarily align exactly. The iWAT uptake (arrowheads) is seen as hot spots bilateral to the bladder and is stronger for KO. The bladder uptake (bottom midline) is due to concentration and excretion of FDG and is expected. *P*-value is determined by two-tailed Student's *t*-test unless otherwise indicated.

To determine the possible involvement of *Nnat* in thermogenesis *in vivo*, we obtained global *Nnat* knockout (KO) mice. We confirmed deletion of NNAT in iWAT, eWAT and brain where the NNAT protein is expressed (Figure 2A). KO of *Nnat* has no significant effect on the UCP1 expression in iWAT and BAT compared to wild-type (WT) when mice are housed at 22 °C (Figure 2A). Deletion of *Nnat* did not change the expression of *Blcap* [24], a neighboring gene whose intron lies on the opposite strand of the *Nnat* gene (Supplementary Figure 2A). We assessed energy metabolism and body composition in WT and KO littermates. Relative to WT, the *Nnat* KO mice fed regular diet did not differ significantly in body weight (25.09 ± 0.34 g vs. 24.22 ± 0.67 g, *p* = 0.29) but showed a trend for a lower lean mass (19.92 ± 0.20 g vs. 18.84 ± 0.46 g, *p* = 0.06). There were no disparities in the respiratory exchange ratio, food intake or physical activity at different temperatures (Supplementary Figs. 2B–D). When energy expenditure was plotted relative to lean mass [25], a genotype effect independent of lean mass could be seen (Figure 2B–D). Analysis of covariance (ANCOVA) with lean mass as a covariate demonstrated significantly higher energy expenditure for the *Nnat* KO mice at 30 °C (*p* = 0.01) and 22 °C (*p* = 0.05) and a trend at 10 °C (*p* = 0.06). Dark cycle energy expenditure was increased in the KO mice at each temperature (*p* = 0.04, 0.004, and 0.02) (Supplementary Figs. 2E–G). The WT and KO mice showed similar amounts of food intake when followed over a longer period of 12 days at 22 °C and exhibited no differences in glucose tolerance (Supplementary Figs. 2H and I).

We next examined thermoregulation in the *Nnat* KO mice. We first tested for a potential effect of the genotype on cold tolerance by acclimating both WT and KO mice at 30 °C for 4 weeks and exposing them to mild cold at 22 °C. As shown in Figure 2E, the *Nnat* KO mice were more tolerant to acute cold challenge and maintained their core body temperature better compared to WT. We observed similar patterns of cold tolerance when we kept the mice at 22 °C and challenged them to severe cold at 7 °C (Figure 2F). We also directly measured the tissue temperatures following CL316,243 treatment using implanted thermocouple probes and found that the iWAT temperature of the *Nnat* KO mice increased at a higher rate than WT (Figure 2G). The BAT temperature changes did not differ significantly between WT and KO (Supplementary Figure 2J). [^18^F]Fluorodeoxy-glucose (FDG)-positron emission tomography (PET) imaging is used to detect thermogenic adipose tissue, with the increase in BAT FDG uptake correlating positively with changes in energy expenditure in humans and mice [20,[25], [26], [27]]. On FDG-PET imaging, we noted a significant increase in CL316,243-stimulated FDG uptake in iWAT for KO mice compared to WT (Figure 2H,I), consistent with a higher thermogenic activity in KO iWAT. In contrast, CL316,243-stimulated uptake in BAT did not differ significantly between KO and WT BAT (Supplementary Fig, 2K and L). The combination of both enhanced adrenergic-stimulated iWAT temperature rise and higher iWAT FDG uptake in the *Nnat* KO mice suggests an elevated thermogenic capacity in iWAT, which can contribute to the better cold tolerance of the KO animals.

To determine if deletion of *Nnat* triggers activation of the thermogenic gene expression program, we examined the regulation of thermogenic and beige fat marker genes. Although short interfering RNA (siRNA)-mediated depletion of *Nnat* in primary adipocytes was reported to induce thermogenic genes [28], we observed no significant differences between WT and KO iWAT (Supplementary Figure 3A) or between beige adipocytes cultured from the WT and KO iWAT stromal vascular fraction (Supplementary Figure 3B). No differences were noted between WT and KO in the histological appearance of iWAT or lipid droplet accumulation as assessed by Oil Red O staining (Supplementary Figs. 3C and D). This suggests that deletion of *Nnat* is not sufficient by itself to drive the morphological changes associated with iWAT beiging but utilizes other mechanisms to enhance the thermogenic capacity.

### 3.3 NNAT inhibits adipocyte thermogenesis in a cell autonomous fashion

**Figure 3:**
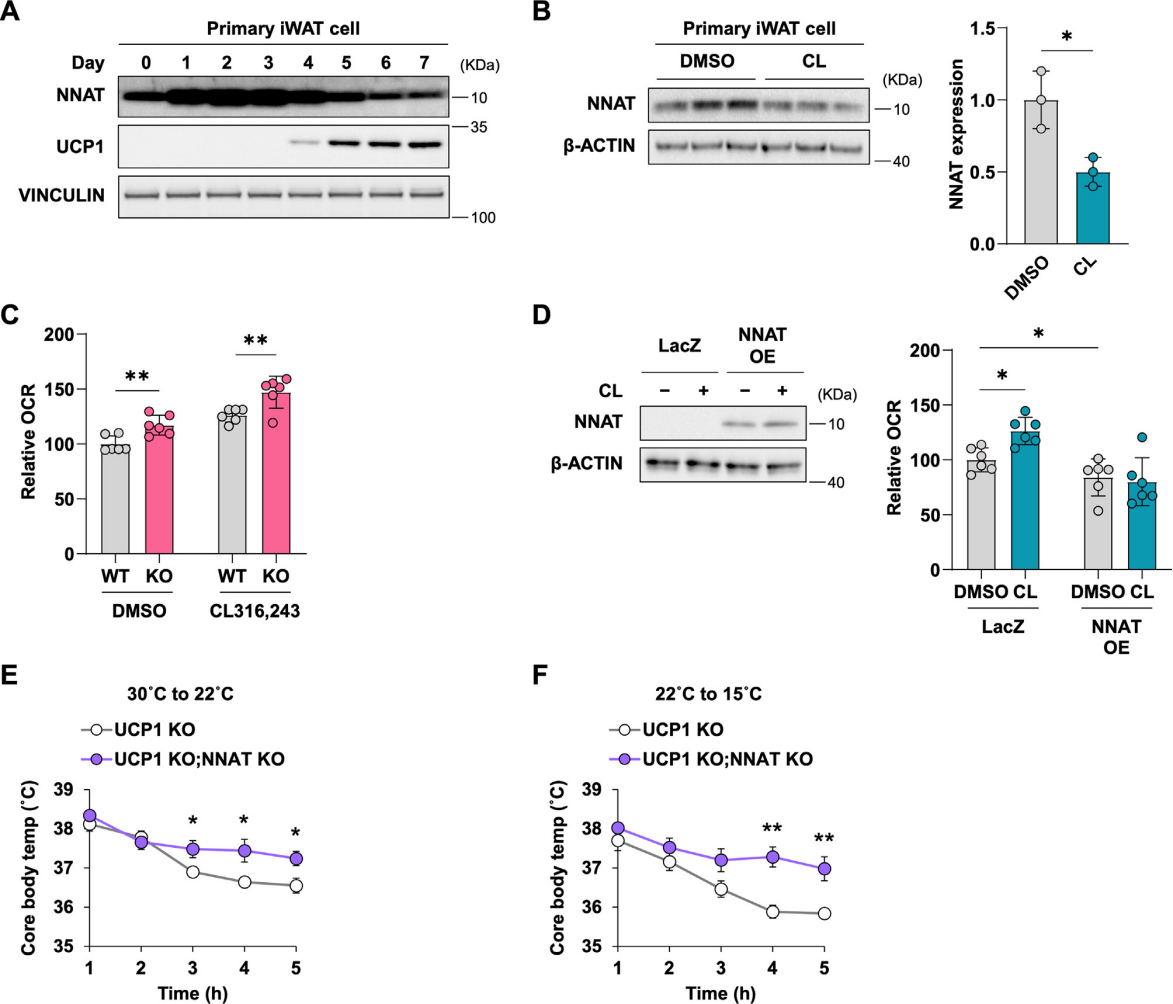
**NNAT controls the thermogenic capacity of beige adipocytes in a UCP1-independent manner** (A) NNAT protein level during differentiation of primary iWAT cells to beige adipocytes. (B) NNAT protein level after the treatment with 10 μM CL316,243 for 48 h in primary iWAT cells. (C) OCR in the iWAT cells derived from *Nnat* KO or WT-littermate in the absence (DMSO only) or presence of 10 μM CL316,243. (D) OCR in iWAT cells derived from WT mice and ectopically expressing *LacZ* or *Nnat* OE with or without treatment with 10 μM of CL316,243. An immunoblot image of NNAT expression is shown. (E and F) Effect of acute cold exposure from 30 °C to 22 °C (E) and that from 22 °C to 7 °C (F) on core rectal temperature of *Ucp1* KO and the *Ucp1*/*Nnat* double KO mice. n = 5 for both groups. *P*-value is determined by two-tailed Student's *t*-test.

To assess whether the adipose tissue phenotype in the *Nnat* KO mice is cell-autonomous, we employed stromal vascular cells isolated from iWAT and differentiated them to beige adipocytes that express thermogenic markers such as Ucp1 [29]. The NNAT protein level peaked during early differentiation (Figure 3A). We observed a reduction in NNAT expression following treatment with the β3-adrenergic agonist CL316,243 in primary iWAT cells (Figure 3B), mirroring the suppression of NNAT in adipose tissue *in vivo* by administration of CL316,243 (Figure 1H,I). We then measured the oxygen consumption rate (OCR) in differentiated primary iWAT cells. Differentiated primary iWAT cells derived from the *Nnat* KO mice showed enhanced oxygen consumption compared to those from WT in the absence and presence of CL316,243 (Figure 3C), in accordance with the enhanced thermogenesis observed in *Nnat* KO mice *in vivo* (Figure 2). In contrast, in brown adipocytes, deletion of *Nnat* KO did not appreciably affect oxygen consumption compared to WT (Supplementary Figure 3E). Next, we examined the gain-of-function effect of *Nnat* on the thermogenesis of beige adipocytes. We demonstrated that *Nnat* overexpression (OE) abolished the CL316,243-mediated increase in oxygen consumption in beige adipocytes (Figure 3D). Nnat OE cells exhibited lower oxygen consumption, both basal and CL316,242-stimulated.

In beige adipocytes, multiple mechanisms of UCP1-independent thermogenesis have been reported, whereas thermogenesis in brown adipocytes is dominantly mediated by UCP1 [30]. To determine whether the physiological effects of NNAT are UCP1-dependent, we tested the cold tolerance of the *Ucp1*/*Nnat* double KO compared to the *Ucp1* KO mice. Lowering the ambient temperature revealed that the double KO mice were better able to maintain their core body temperature than their *Ucp1* KO littermates (Figure 3E,F). This suggests that the effects of Nnat on thermal regulation are at least in part independent of UCP1.

### 3.4 Calcium cycling via SERCA2 mediates enhanced thermogenesis in NNAT KO adipocytes

**Figure 4:**
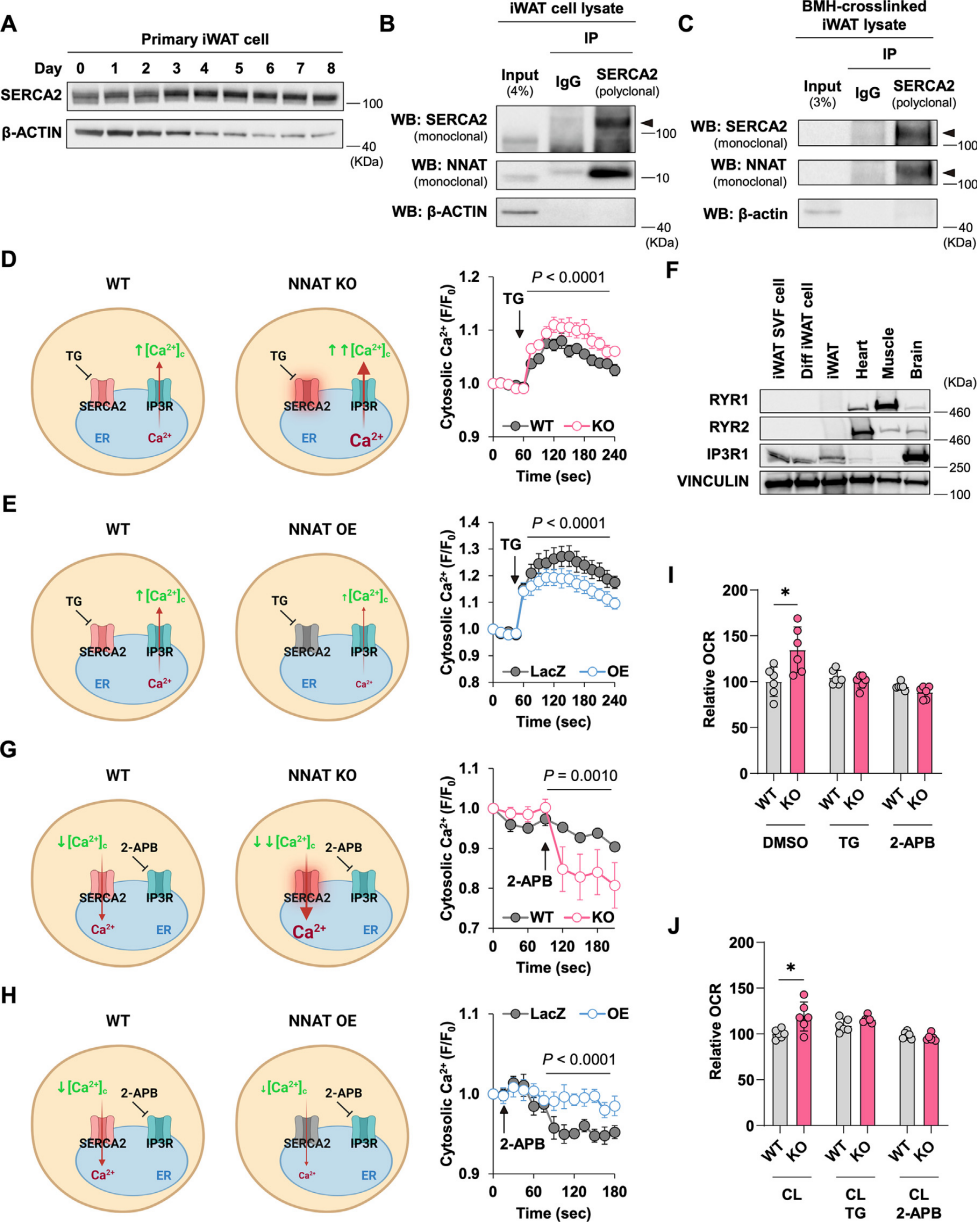
**NNAT regulates calcium cycling and thermogenic respiration via SERCA2** (A) SERCA2 protein levels during differentiation of primary iWAT cells to beige adipocytes. (B and C) Immunoblot of SERCA2 and NNAT after immunoprecipitation by SERCA2 or control IgG in non-crosslinked iWAT cell lysate (B) or crosslinked iWAT lysate (C). For (C), two same sample sets were loaded in different lanes and one was probed with SERCA2 monoclonal antibody and the other with NNAT monoclonal antibody above 100 kDa. (D) Change in the intracellular calcium levels in WT and *Nnat* KO iWAT cells after treatment with 2 μM of thapsigargin (TG). WT, n = 8; *Nnat* KO, n = 9.(E) Change in the intracellular calcium levels in LacZ control and *Nnat* OE iWAT cells after treatment with 2 μM of TG. LacZ, n = 5; *Nnat* OE, n = 5. (F) Protein expression of Ryanodine receptor 1 (RYR1), Ryanodine receptor 2 (RYR2), and IP3 receptor isoform 1 (IP3R1) in the stromal vascular fraction (SVF) of iWAT, differentiated iWAT cells, and indicated tissues. (G) Change in the intracellular calcium levels in WT and *Nnat* KO iWAT cells after treatment with 50 μM of 2-aminoethyl diphenylborinate (2-APB). WT, n = 10; *Nnat* KO, n = 10. (H) Change in the intracellular calcium levels in LacZ control and *Nnat* OE iWAT cells after treatment with 50 μM of 2-APB. LacZ, n = 6; *Nnat* OE, n = 6. (I and J) OCR in WT and *Nnat* KO iWAT cells treated with 2 μM TG or 50 μM 2-APB for 30 min in the absence (I) or presence of 10 μM CL316,243 (J). At least biological triplicates were used. *P*-value is determined by two-tailed Student's *t*-test (I, J) or two-way ANOVA followed by Fisher's LSD test (D, E, G, H).

A recently proposed mechanism for UCP1-independent thermogenesis in beige fat involves SERCA2-mediated calcium cycling [9]. NNAT has significant sequence homology to two known SERCA binding proteins, phospholamban and sarcolipin, which control ER/SR calcium transport through modulation of SERCA activity [[14], [15], [16]]. Phospholamban limits the affinity of SERCA for Ca^2+^ in a β-adrenergic signaling-dependent manner and is key to the cardiac component of the fight-or-flight response [14]. Sarcolipin has been implicated in the uncoupling of the SERCA pump and UCP1-independent thermogenesis in skeletal muscle [15, 16]. The phospholamban and sarcolipin mRNA levels in iWAT cells and tissues are not regulated by β3-adrenergic stimulation or by deletion of *Nnat* (Supplementary Figs. 4A and B). NNAT has previously been reported to inhibit SERCA activity in neurons and embryonic stem cells undergoing neuronal differentiation [13, 31, 32]. These considerations raised the possibility that a functional interaction between NNAT and SERCA can modulate adipocyte thermogenesis. We found that SERCA2, the predominant isoform of SERCA in iWAT [9], increases in expression during iWAT differentiation (Figure 4A) and physically binds NNAT in iWAT based on co-immunoprecipitation and crosslinking studies (Figure 4B,C, and Supplementary Figure 4C). Next, we evaluated whether alteration of the NNAT level affects ER Ca^2+^ transport. The ER Ca^2+^ stores are largely determined by the influx via SERCA and the efflux via the Ca^2+^ release channels. Enhanced SERCA-mediated Ca^2+^ transport into the ER will increase the steady-state ER Ca^2+^ loading in *Nnat* KO cells. To estimate the ER Ca^2+^ stores, we treated adipocytes with thapsigargin (TG), a non-competitive SERCA inhibitor, and monitored cytosolic Ca^2+^ concentrations with the Ca^2+^ indicator Fluo-4 AM. The TG-induced cytosolic Ca^2+^ increase from the release of the ER Ca^2+^ stores is transient, lasting minutes, because excess cytosolic Ca^2+^ can be taken up into mitochondria or move across the plasma membrane via Na ^+^ -Ca^2+^ exchangers. The *Nnat* KO cells showed a significantly larger magnitude of Ca^2+^ release from the ER to the cytosol following TG treatment (Figure 4D), consistent with a higher baseline SERCA pump activity and greater ER Ca^2+^ stores in the KO cells. Conversely, in the *Nnat* OE cells, the Ca^2+^ efflux from the ER was diminished compared to LacZ control, indicating a reduced steady-state ER Ca^2+^ load and decreased SERCA activity (Figure 4E). Ca^2+^ cycling involves the activity of Ca^2+^ release channels as well as the SERCA pump activity. We examined the expression of several known Ca^2+^ release channels and found that inositol 1,4,5-trisphosphate receptor type 1 (IP3R1) was highly expressed in iWAT and primary iWAT cells (Figure 4F). The IP3R1 conductance rises with higher IP_3_ concentrations but a substantial percentage of these channels can already be open under unstimulated conditions (∼100 nM IP_3_) [33], allowing continuous leakage of Ca^2+^. Blocking the IP3R activity with 2-APB, an IP3R inhibitor, lowered the cytosolic Ca^2+^ more in the *Nnat* KO cells than in 10.13039/100010269WT cells (Figure 4G), which supports enhanced SERCA activity and also confirms the importance of IP3R as a leak channel in this context. The *Nnat* OE cells, on the other hand, exhibited higher cytosolic Ca^2+^ levels compared to LacZ cells following 2-APB treatment (Figure 4H), suggesting reduced baseline SERCA activity. We also employed IP3R activators such as cell-permeable, photoactivatable IP3 (ci-IP3/PM) and phospholipase C activators, in *Nnat* OE HeLa cells and noted a reduction in the Ca^2+^ release from the ER, again consistent with lower ER Ca^2+^ loading in *Nnat* OE cells (Supplementary Figs. 4D and E). Altogether, our observations demonstrate that NNAT inhibits SERCA2 activity and attenuates ER Ca^2+^ cycling in adipocytes.

We also assessed the extent to which the increased thermogenic respiration in the *Nnat* KO adipocytes is dependent on these alterations of Ca^2+^ flux. The WT and the *Nnat* KO iWAT cells were treated with TG or 2-APB in the absence and presence of CL316,243. We found both TG and 2-APB treatment abolished the enhancement of the OCR in the *Nnat* KO cells with or without CL316,243 (Figures 4I,J), indicating that Ca^2+^ entry into and exit from the ER are required for the increase in thermogenic respiration. These observations support the notion that modulation of Ca^2+^ cycling by NNAT is key to its role in adipocyte thermogenesis.

### 3.5 Cold exposure induces NHLRC1, which targets the NNAT protein for degradation

We next sought to investigate the basis for acute regulation of NNAT expression in response to cold stimuli. To serve as a physiologically important modulator of NNAT expression in the context of cold-induced thermogenesis, the upstream regulator itself has to exhibit sensitivity to cold stress. Several studies have noted an inverse correlation between the expression levels of *mir-708-5p* and *Nnat* in the brain and pancreatic β-cells, and this miRNA targets the 3′-untranslated region of the *Nnat* mRNA for degradation [32, 34]. However, when we tested whether the *mir-708-5p* level is altered in response to cold in iWAT, neither acute cold nor chronic cold significantly affected the miRNA expression (Supplementary Figure 5A).

**Figure 5:**
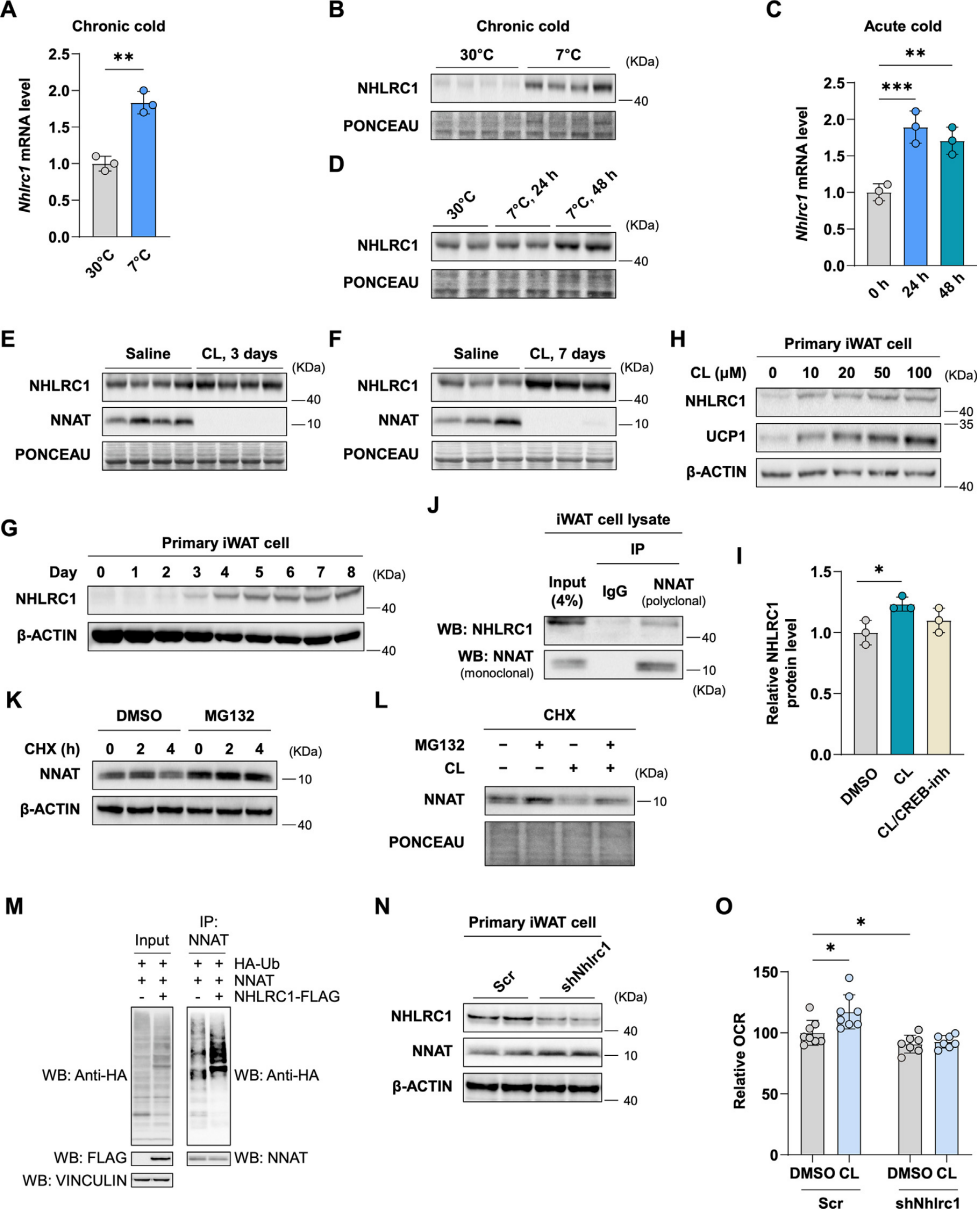
**The E3 ubiquitin ligase NHLRC1 is induced by cold and regulates NNAT degradation** (A) NHLRC1 protein level during differentiation of primary iWAT cells to beige adipocytes. (B to E) The mRNA and protein expression of NHLRC1 in iWAT after chronic cold (B, C) and acute cold exposure (D, E). (F and G) NHLRC1 protein level in iWAT of mice injected with saline or CL316,243 for 3 days (F) or 7 days (G). (H) The protein levels of NHLRC1 in differentiated primary iWAT cells treated with varying concentrations of CL316,243 for 24 h. (I) The relative protein levels of NHLRC1 in differentiated primary iWAT cells treated with DMSO, 10 μM of CL316,243, or 10 μM of CL316,243 + 1 μM of CREB-inhibitor (666–15, Sigma). (J) Immunoblot of NHLRC1 and NNAT after immunoprecipitation by NNAT or control IgG in MG132-treated iWAT cell lysate. (K) In the absence or presence of MG132, iWAT cells were treated with cycloheximide (CHX) and harvested at the indicated time. (L) Differentiated iWAT cells were treated with either DMSO or MG132 for 4 h before the addition of CHX and CL316,243. Cells were harvested at 6 h post CHX treatment. (M) NHLRC1 mediates ubiquitination of NNAT. 293FT cells were transfected with plasmids encoding HA-Ubiquitin and NNAT, along with NHLRC1-FLAG or empty-FLAG and incubated for 48 h. After treatment with 10 μM MG132 for 6 h, cell lysate was prepared and used for IP using polyclonal NNAT antibody. Input (9%) and IP samples were immunoblotted with anti-HA antibody. Vinculin was used as a loading control. (N) NHLRC1 and NNAT protein levels in differentiated iWAT cells expressing a scrambled shRNA (control) or the *Nhlrc1* shRNA. (O) OCR in differentiated iWAT cells expressing a scrambled RNA or the *Nhlrc1* shRNA in the presence and the absence of 10 μM CL316,243. *P*-value is determined by two-tailed Student's *t*-test.

It has been reported that NHLRC1, an E3 ubiquitin protein ligase, mediates NNAT ubiquitination in PC12 neuroendocrine tumor cells [35]. We thus sought to examine a possible role for NHLRC1 in regulating NNAT in adipose tissue. NHLRC1 is readily detected in iWAT and multiple other tissues (Supplementary Figs. 5B and C). The *Nhlrc1* mRNA and protein levels correlate negatively with the changes in the *Nnat* expression in a number of different settings, including iWAT tissue in mice subjected to chronic or acute cold exposure (Figure 5A–D), and iWAT tissue in mice treated for short-term or long-term with a β3-adrenergic agonist (Figure 5E,F). NHLRC1 increased during later stages of beige adipocyte differentiation (Figure 5G). Consistent with the induction of NHLRC1 by cold exposure and β3-adrenergic stimulation *in vivo*, primary iWAT cells treated with varying concentrations of CL316,243 also increased NHLRC1 (Figure 5H), and this increase was abrogated when cells were simultaneously treated with an inhibitor of CREB, the cAMP-responsive transcription factor (Figure 5I). The inverse correlation between NNAT and NHLRC1 expression led us to probe proteasome-mediated degradation of NNAT in beige adipocytes. We found that NNAT physically interacted with NHLRC1 (Figure 5J) and the treatment with a proteasome inhibitor, MG132, attenuated NNAT degradation in iWAT cells (Figure 5K). Moreover, the β3-adrenergic agonist-induced decrease in NNAT levels was rescued by MG132 treatment (Figure 5L), supporting proteasome-dependent degradation of the NNAT protein in cold-stimulated adipose tissue.

In co-transfection experiments in cells expressing endogenous or exogenous *Nnat*, titrating up the amount of transfected *Nhlrc1* resulted in a reduction in the NNAT protein level (Supplementary Figs. 5D and E). NHLRC1 increased NNAT ubiquitination (Figure 5M). We also tested the functional importance of NHLRC1 in NNAT degradation by lentiviral shRNA-mediated depletion of NHLRC1. We observed that iWAT cells expressing the *Nhlrc1* shRNA expressed higher levels of NNAT proteins relative to those expressing the control shRNA (Figure 5N). Furthermore, the CL316,243-mediated increase in OCR was blocked when the NHLRC1 level was reduced by the *Nhlrc1* shRNA (Figure 5O), similar to the effect of *Nnat* OE on respiration (Figure 3D). Taken together, these data suggest that NHLRC1 plays a key role in linking environmental stimuli such as cold exposure or β3-adrenergic stimulation to a regulated decrease in the NNAT protein amount in iWAT, which in turn controls SERCA function and thermogenesis.

## 4 Discussion

We have described here a novel regulator of thermogenesis, NNAT, which interacts with SERCA2 to control ER Ca^2+^ cycling in adipocytes. The NNAT levels in adipose tissues undergo regulation by temperature changes or adrenergic stimulation (Figure 1), and loss of *Nnat* enhances the thermogenic capacity in iWAT tissues (Figure 2) and cells (Figure 3). We initially focused our attention on *Nnat* after noting in transcriptome analysis that it is the most strongly suppressed gene in white fat after cold exposure (Figure 1A). A number of additional features were suggestive of the potential involvement of *Nnat* in the regulation of energy homeostasis. *Nnat* is imprinted and is transcribed only from the paternal allele [24]. Imprinted genes are frequently involved in controlling processes essential to the development of fetal and newborn animals, such as growth, feeding, and thermoregulation [36]. Our data indicate that *Nnat* is highly expressed in white adipose tissue while being very low in thermogenic brown adipose tissue (Figure 1B,C). Previous studies found that a single nucleotide polymorphism of the human *Nnat* gene is associated with obesity in children and adults [37] and paternal obesity is linked to hypomethylation at the *Nnat* differentially methylated regions (DMRs) in the offspring [38]. *Nnat* is also detected in pancreatic β-cells, where it has a role in glucose-stimulated insulin secretion and its own expression is glucose-regulated [12]. Such regulation of the *Nnat* expression levels in altered metabolic states suggests that *Nnat* may have a role in energy homeostasis. More recently, it has been suggested that *Nnat* regulates thermogenesis in muscle cells [17,18]. However, *Nnat* is expressed at the highest level in WAT (Figure 1C), a key metabolic organ. Here, we provide evidence that *Nnat* has an inhibitory effect on adipocyte thermogenesis, and suggest that a regulated decrease in this protein upon cold exposure is a key step.

The NNAT protein levels are suppressed by β3-adrenergic stimulation both in cultured primary iWAT cells (Figure 3B) and in iWAT tissue *in vivo* (Figure 1D–I), which parallels the activation of thermogenesis in this tissue. Loss of NNAT does not significantly enhance the expression of thermogenic genes or beige fat marker genes in iWAT (Supplementary Figs. 3A and B), suggesting that transcriptional regulation of the conversion of iWAT to beige fat is not the main mechanism by which NNAT acts. Similarly, adipose-selective deletion of SERCA2 impairs iWAT thermogenesis by abrogating Ca^2+^ cycling, without affecting iWAT thermogenic gene expression or histology [9].

In a study of the β-cell phenotype in mice deleted of the paternal *Nnat* allele, no significant body weight or feeding phenotype was found in the C57BL/6J background [12], which is in agreement with our data. A subsequent study by the same group noted two distinct subpopulations among the paternally deleted mice, one having a normal weight and another with relative leanness [39], but this did not result in differences in the average body weight or food intake from the control mice. When studied in the 129S2/Sv background, however, deletion of *Nnat* was associated with a lower body weight early in life and with hyperphagia later in life [39]. Thus, genetic background can modify the body weight phenotype of the *Nnat* deficient mice in a complex fashion. Fully dissecting these effects will likely require the use of conditional mouse models in different genetic backgrounds.

Under a cold challenge, the *Nnat* KO mice were better able to maintain their core body temperature relative to the WT mice (Figure 2E,F), consistent with the idea that NNAT impairs cold adaptation. These KO mice produce greater temperature increases within iWAT tissue upon β3-adrenergic stimulation (Figure 2G) and also exhibit greater FDG uptake in iWAT on PET imaging, indicative of elevated metabolic activity in that tissue (Figure 2H,I). CL316,243 does not cross the blood brain barrier [[40], [41], [42]] and is likely acting primarily on iWAT cells to enhance FDG uptake and heat production. The cell-autonomous effects of NNAT on adipocyte thermogenesis have been demonstrated by measuring thermogenic respiration in the *Nnat* KO iWAT cells and the *Nnat* OE iWAT cells differentiated from primary stromal vascular cells (Figure 3C,D). The *Nnat* KO iWAT cells display increased respiration even without β-adrenergic stimulation (Figure 3C), unlike the UCP1-dependent leak respiration that requires activation with fatty acids. In addition, the observation that the *Ucp1/Nnat* double KO mice exhibit greater cold tolerance than their Ucp1 KO littermates (Figure 3E,F) suggest UCP1-independence of the effects of NNAT on thermogenesis.

Our data in this study point to a role for ER Ca^2+^ cycling in NNAT action. We have presented evidence that the effects of *Nnat* deletion on adipocyte thermogenic respiration are dependent on Ca^2+^ flux via the ER Ca^2+^ pump SERCA2 (Figure 4). The physical interaction between NNAT and SERCA2 in adipocytes (Figure 4B,C) likely represents a key event in the regulation of iWAT Ca^2+^ cycling and thermogenesis. NNAT has structural similarities to phospholamban, a small membrane protein that binds and inhibits SERCA2 in cardiac muscle [43]. Dissociation of phospholamban from SERCA2 results in full activation of SERCA, promoting Ca^2+^ uptake into the SR and cardiac muscle relaxation [14]. Phospholamban is thus critical for cardiac function. Superinhibitory phospholamban gene mutations manifest in dilated cardiomyopathy, while loss-of-function mutations are also linked to heart failure [14, [44], [45], [46]]. Another small SR membrane protein, sarcolipin, uncouples SERCA ATPase activity from Ca^2+^ transport in skeletal muscle and mediates UCP1-independent thermogenesis in that tissue [16, 47]. While phospholamban and sarcolipin both bind SERCA and share sequence similarities, their physiological roles are distinct due to tissue expression and differential effects on SERCA activity. How NNAT alters SERCA function will likely impact the physiological consequences of its loss. Our data suggest that deletion of *Nnat* increases the Ca^2+^ flux through SERCA and also through IP3R. When Ca^2+^ exit from the ER is blocked, the intracytosolic Ca^2+^ level declines more precipitously in the *Nnat* KO iWAT cells, reflecting greater Ca^2+^ transport by SERCA (Figure 4G). Blocking Ca^2+^ entry into the ER, on the other hand, uncovers a greater magnitude of Ca^2+^ efflux into the cytosol in the KO iWAT cells (Figure 4D). Increased thermogenesis can be achieved by raising the quantity of ATP hydrolyzed per Ca^2+^ cycle or by enabling greater cycling of Ca^2+^ even if the amount of heat produced per Ca^2+^ cycle stays the same.

There are examples in nature of futile Ca^2+^ cycling causing increased tissue temperatures. Ocular heater organs in some deep sea fish species continuously take up Ca^2+^ via SERCA and release Ca^2+^ via ryanodine receptors (RyRs) to warm the brain and eyes up to 14 °C above ambient water temperature [48]. Inherited mutations in the human ryanodine receptor 1 (RYR1) gene produce excess Ca^2+^ release from the SR and hyperthermia when exposed to triggering agents [49]. A recent study reported that deletion of SERCA2b impaired UCP1-independent beige fat thermogenesis in mouse beige adipocytes [9]. shRNA-mediated depletion of SERCA2b reduced norepinephrine-induced respiration in pig adipocytes, which lack functional UCP1 [9].

**Figure 6:**
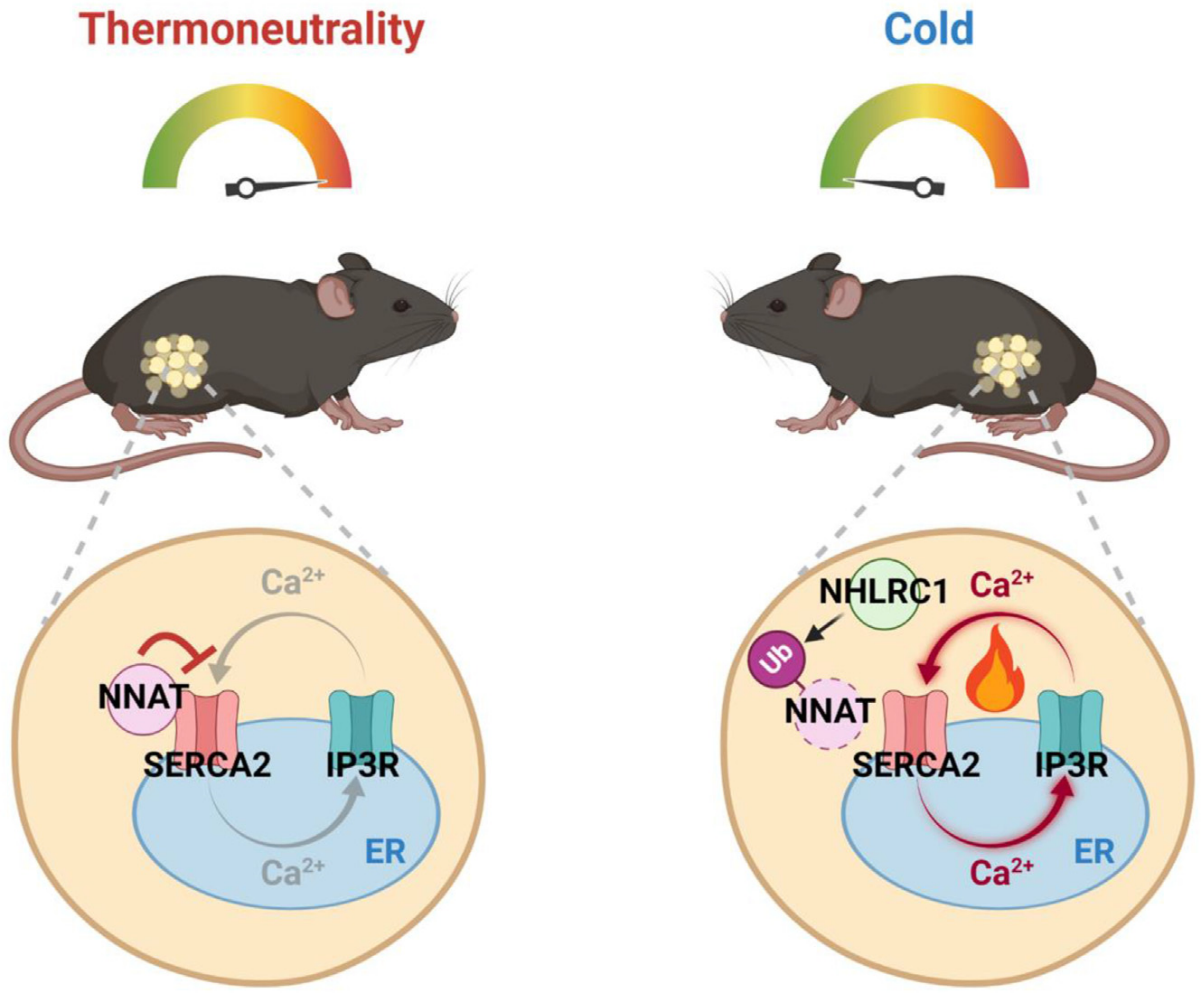
**Overview of functional and physical interaction between NNAT and SERCA**. A schematic model of regulation of noncanonical thermogenesis by NNAT in adipocytes. At thermoneutrality, NNAT interacts with SERCA2 and inhibits calcium cycling. Cold exposure or treatment with a β3-adrenergic agonist increases intracellular cAMP levels, which induces NHLRC1 expression. The E3 ubiquitin ligase NHLRC1 then ubiquitinates and degrades NNAT, leading to activation of SERCA2. The futile calcium cycling mediated by SERCA2 dissipates energy as heat.

In our model, depicted in Figure 6, NNAT inhibits ER Ca^2+^ cycling via its interactions with SERCA2b, the predominant isoform of SERCA in adipocytes. Upon release from inhibition by NNAT, SERCA2b mediates increased Ca^2+^ transport into the ER, which is accompanied by increased Ca^2+^ exit back into the cytosol via channels such as IP3R. In the *Nnat* KO adipocytes, the absence of NNAT is predicted to result in a constitutively increased transport of Ca^2+^ into the ER and elevated ER calcium stores at the steady-state. We suggest that NNAT serves a role as the β-adrenergic signaling-responsive regulator of SERCA2b activity in adipose tissue and thereby can modulate UCP1-independent thermogenesis in that tissue. Although *Nnat* is expressed at the highest level in WAT, it is possible that a similar thermogenic mechanism involving NNAT and SERCA is operative in other tissues such as muscle and contributes to systemic energy expenditure [17,18].

We have also demonstrated that, upon cold challenge or treatment with a β3-adrenergic agonist, the E3 ubiquitin ligase NHLRC1 is induced (Figure 5A–H) and this induction involves the activity of the cAMP-responsive transcription factor CREB (Figure 5I). NHLRC1 physically interacts with NNAT (Figure 5J) and mediates the proteasome-dependent degradation of NNAT (Figure 5K to N, and Figure 3B,C), freeing up SERCA2b (Figure 6). This completes a regulatory circuit connecting external thermogenic stimuli to increased Ca^2+^ flux into and out of the ER, translating into energy expenditure.

Limitations of the present study include the use of the global *Nnat* KO mouse model, which does not eliminate possible contributions from NNAT expressed in the brain and at low levels in other tissues. This will be addressed by utilizing conditional mouse models to dissect tissue-selective physiology *in vivo*. Because most small molecules do not cross the blood brain barrier, pharmacological disruption of the interaction between NNAT and SERCA2 may offer a potential strategy to increase energy expenditure with low off-target effects if adipose tissue is the main site of interaction between these two proteins.

## Author contributions

K.C. and J.C.Y. conceived the study and designed experiments. K.C. performed most of the experiments and C.Y.K., S.M.A., S.H.C., and J.H.K. assisted with experiments. D.J.R. analyzed the PET imaging data. K.C., C.Y.K., S.M.A., S.H.C., D.J.R., J.H.K., A.F., A.J.C., D.M.B. and J.C.Y. interpreted the data. K.C. and J.C.Y. wrote the manuscript. K.C., C.Y.K., A.F., D.M.B. and J.C.Y. edited the manuscript.

## Data Availability

The RNA-seq data were deposited to Gene Expression Omnibus in the accession number of GSE133619.
