# Supplementary material for: Regulation of beige adipocyte thermogenesis by the cold-repressed ER protein NNAT
Source: Mol Metab. 2023 Jan 25;69:101679. doi: 10.1016/j.molmet.2023.101679 (PMC9932177; doi:10.1016/j.molmet.2023.101679)
Supplement: Multimedia component 1 [file mmc1.pdf]

Supplementary Information

Regulation of beige adipocyte thermogenesis by the cold-repressed ER protein NNAT

Kyung-Mi Choi, Christopher Y. Ko, Sung-Min An, Seung-Hee Cho, Douglas J. Rowland, Jung Hak Kim, Anna Fasoli, Abhijit J. Chaudhari, Donald M. Bers, John C. Yoon

Supplementary Table 1. Primers used in this study

Primer	Forward (5' to 3')	Reverse (5' to 3')	Purpose
Nnat WT	GAATTTGTAGGCTTGGGTGTGTCG	GGTACACAGGGATTGCGGCAAT	Genotyping
Nnat KO	GAATTTGTAGGCTTGGGTGTGTCG	CGATCTTCATGGTAGGATCTTGTGC	Genotyping
Nnat CDS	ACCATGGCCGCAGTGGCAG	TCAGTTGGGGGCTCGCTG	Nnat cloning
Nhlrc1 CDS	GCAATGGGGGAGGAGGCGAC	CCATCACCCTCCGTTGCCCT	Nhlrc1 cloning
Nnat	ATCGGCTGGTACATCTTCCG	TGAACACCTCACTTCTCGCA	qPCR
Nhlrc1	CCAGAACTCCAAATCCTGAGAA	GGGAGAGAGAGAGAACACACTA	qPCR
Blcap	ACCAGGAGCTTCCTCATTGCTG	ACACTTGTTTCTGAAGCGCAG	qPCR
PLN variant 1	GCTTCATGCTCTGCACTGTG	TTGTGCAGACTGAAGCGTCA	qPCR
SLN	GGTGGAGAGACTGAGGTCCT	GCACACCAAGGCTTGTCTTC	qPCR
β-Actin	CTAAGGCCAACCGTGAAAAG	ACCAGAGGCATACAGGGACA	qPCR

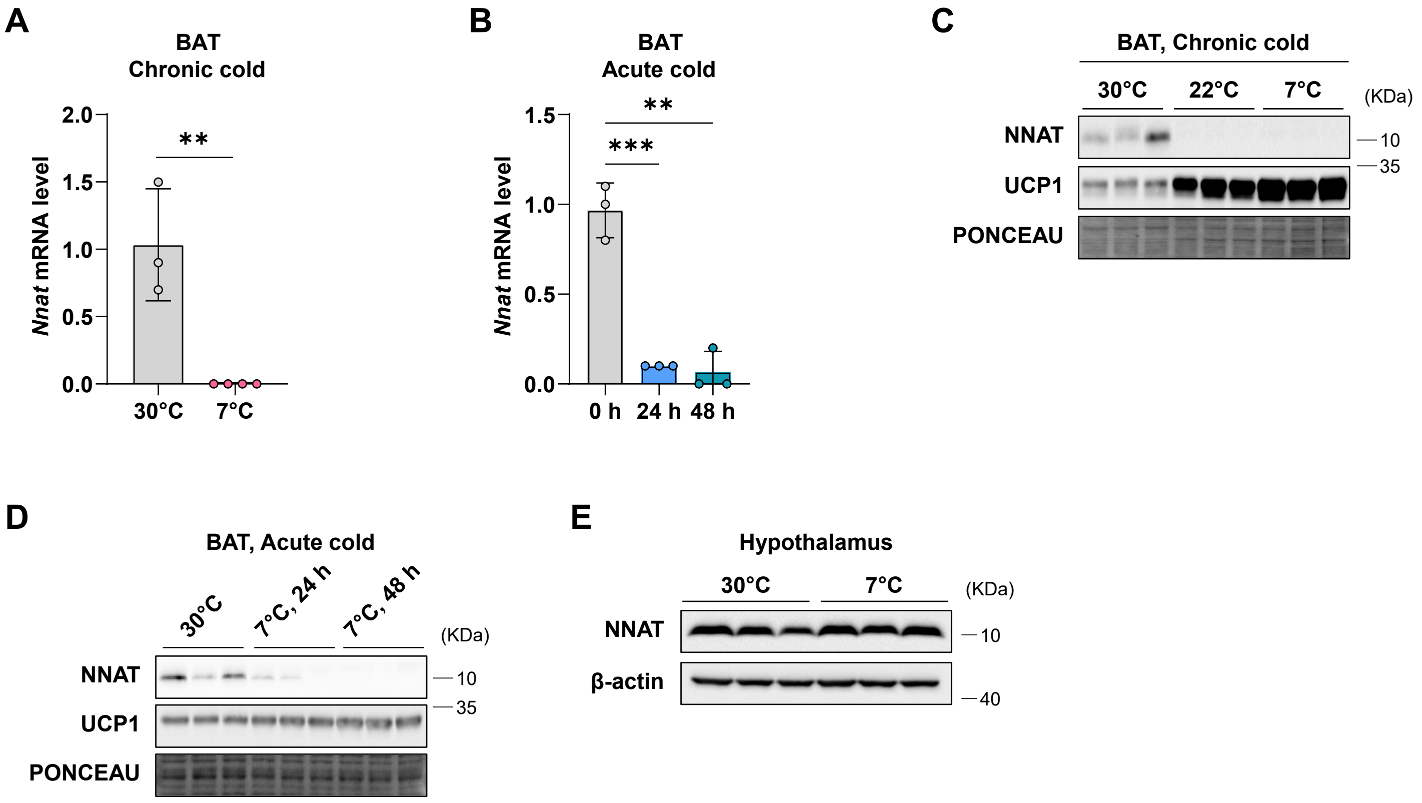

Supplementary Figure 1.

(A) The mRNA level of *Nnat* in BAT after chronic cold exposure. 30ºC, n = 3; 7ºC, n = 4.

(B) The expression of *Nnat* in BAT after acute cold exposure determined by qPCR, n = 3 each.

(C) BAT expression of NNAT determined by immunoblotting under 30ºC, 22ºC, or 7ºC conditions for 3 weeks. Ponceau S staining was used as a loading control.

(D) The NNAT protein expression in BAT after acute cold exposure.

(E) The NNAT protein level in hypothalamus after chronic cold.

Bars are expressed as mean ± SD. *P*-value is determined by two-tailed Student's *t*-test; ** $P \leq 0.01$, *** $P \leq 0.001$.

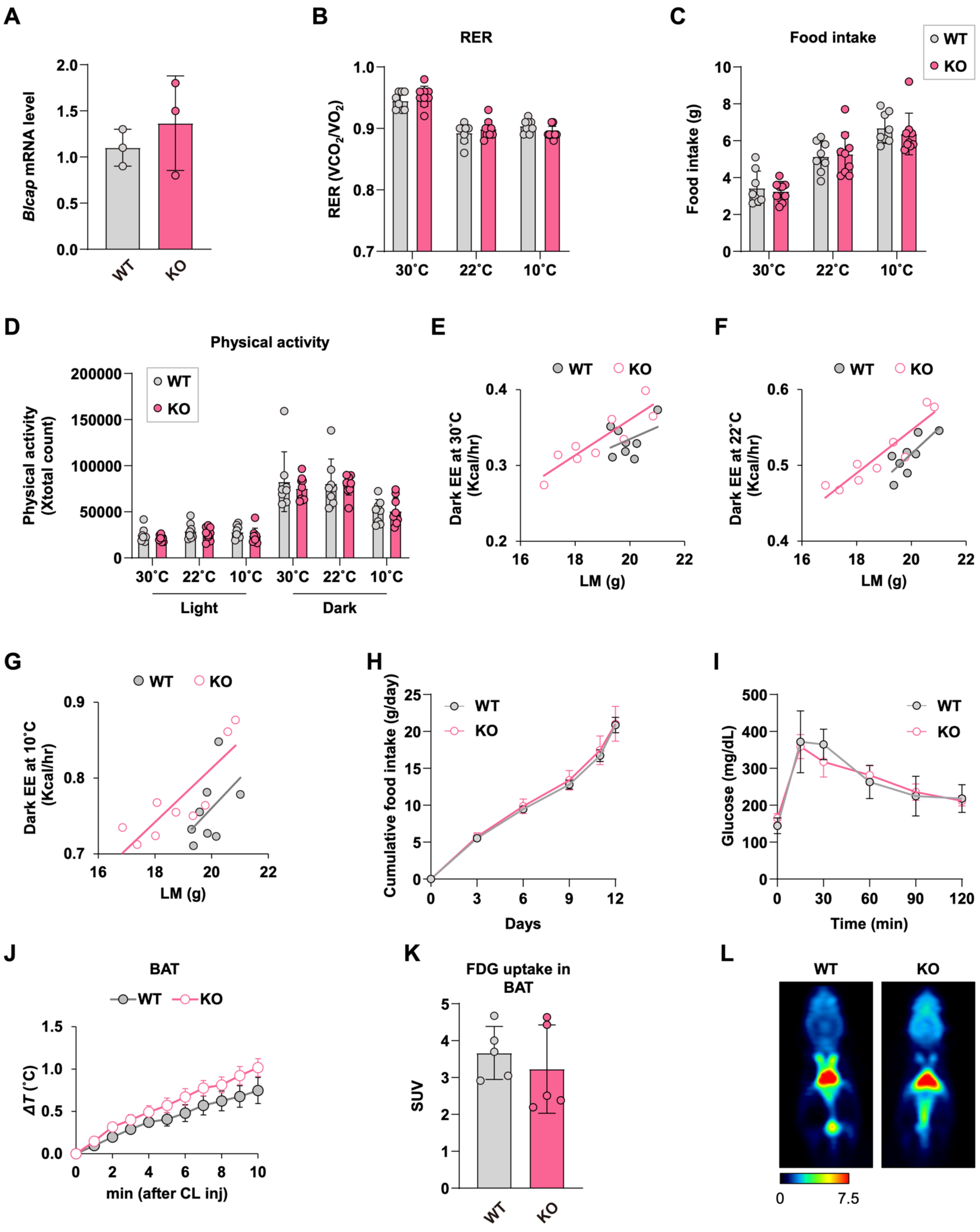

A
Blcap mRNA level
WT
KO
B
RER
RER (VCO2/VO2)
30°C
22°C
10°C
C
Food intake
Food intake (g)
D
Physical activity
Physical activity (Xtotal count)
Light
Dark
E
Dark EE at 30°C (Kcal/hr)
LM (g)
F
Dark EE at 22°C (Kcal/hr)
G
Dark EE at 10°C (Kcal/hr)
H
Cumulative food intake (g/day)
Days
I
Glucose (mg/dL)
Time (min)
J
BAT
ΔT (°C)
min (after CL inj)
K
FDG uptake in BAT
SUV
L
0
7.5

Supplementary Figure 2. Metabolic studies and tissue temperature recording in the *Nnat* KO mouse models

(A) *Blcap* mRNA level in the *Nnat* KO mice versus WT littermate.

(B to D) Comprehensive Lab Animal Monitoring System (CLAMS) experiment in the *Nnat* KO (n = 9) versus WT littermate (n = 8). Respiratory exchange ratio (RER, B) and food intake (C) for 24 h under different temperatures. Physical activity during light and dark cycle under different temperatures (D).

(E to G) Dark cycle energy expenditure (EE) plotted relative to lean mass (LM) and fitted by linear regression.

(H) Cumulative food intake in WT and *Nnat* KO mice over a 2-week period (n = 7 each).

(I) Glucose tolerance test in WT and *Nnat* KO mice (n = 4 each). Mice were fasted overnight and injected with glucose 1 g/kg of body weight by intraperitoneal injection. Blood glucose was monitored at indicated time points using a glucometer.

(J) Changes in BAT temperature following CL316,243 treatment. WT littermate, n = 3; Nnat KO, n = 4.

(K and L) FDG-PET standardized uptake value (SUV) measures for BAT from *Nnat* KO (n = 5) and WT littermate (n = 5) groups (I). A representative image is shown (J). The Mann-Whitney test reveals no significant change in FDG uptake between WT and KO BAT (p = 0.8413).

Bars are expressed as mean ± SD. P-value is determined by two-tailed Student's t-test (A to D, and H to J) or ANCOVA analysis with lean mass as a covariate (E to G). There was no statistical significance.

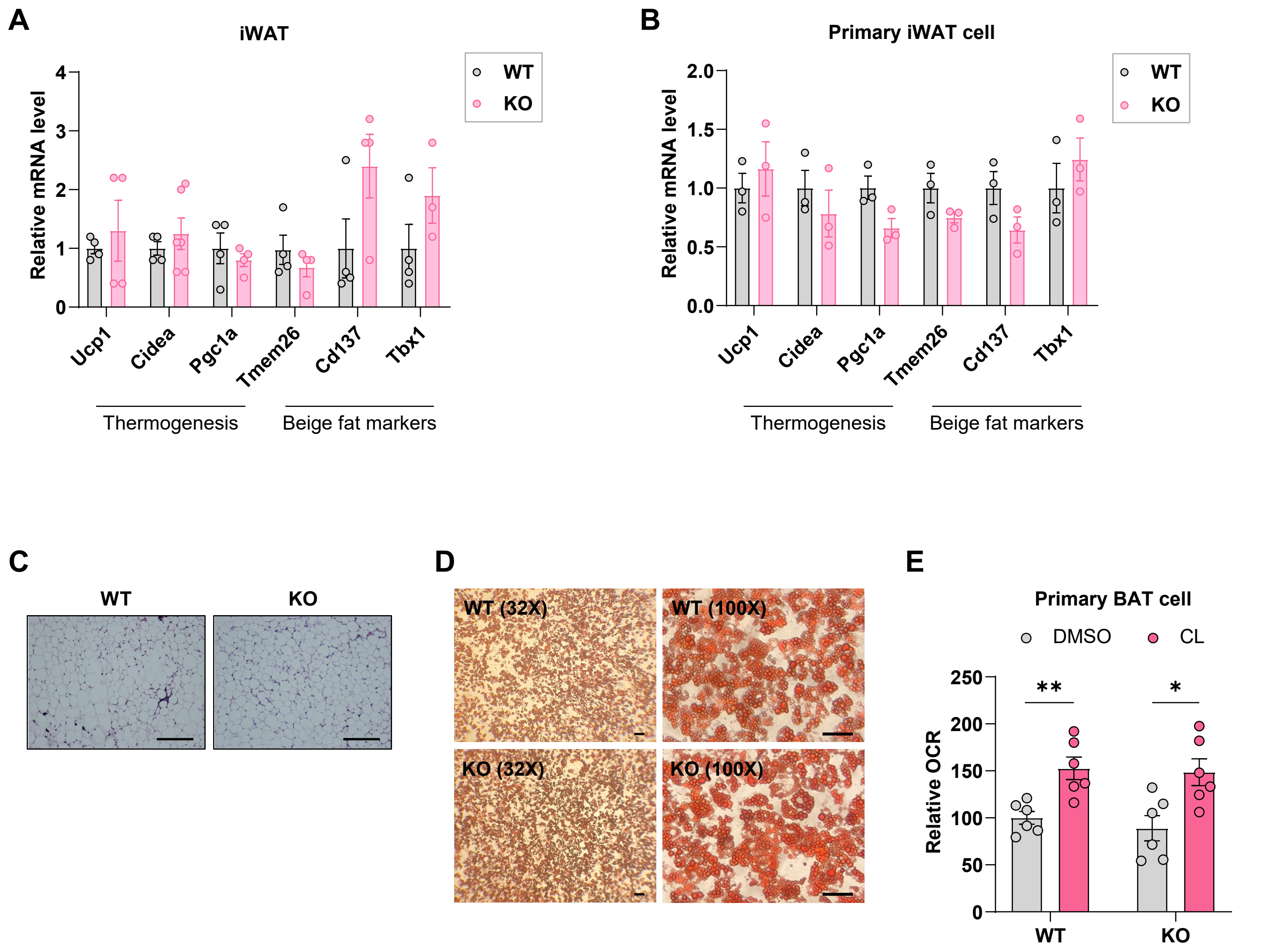

Supplementary Figure 3. Characterization of adipocytes and adipose tissues from WT and *Nnat* KO mice

(A) mRNA expression of thermogenic genes and beige fat marker genes in iWAT of the *Nnat* KO versus WT littermates. n = 3-6 for both groups.

(B) mRNA levels of thermogenic genes and beige fat markers in *Nnat* KO and WT beige adipocytes.

(C) Hematoxylin-eosin (H&E) staining of WT and *Nnat* KO iWAT (scale bar 200 μm).

(D) Representative images of Oil Red O staining in WT and *Nnat* KO beige adipocytes (scale bars 500 μm on the left, 200 μm on the right).

(E) Oxygen consumption rate (OCR) in brown adipocytes derived from *Nnat* KO or WT-littermates in the absence or presence of CL316,243. n = 6 per group.

Bar graphs are represented as mean ± SEM. *P*-value is determined by two-tailed Student's *t*-test; *** $P \leq 0.001$.

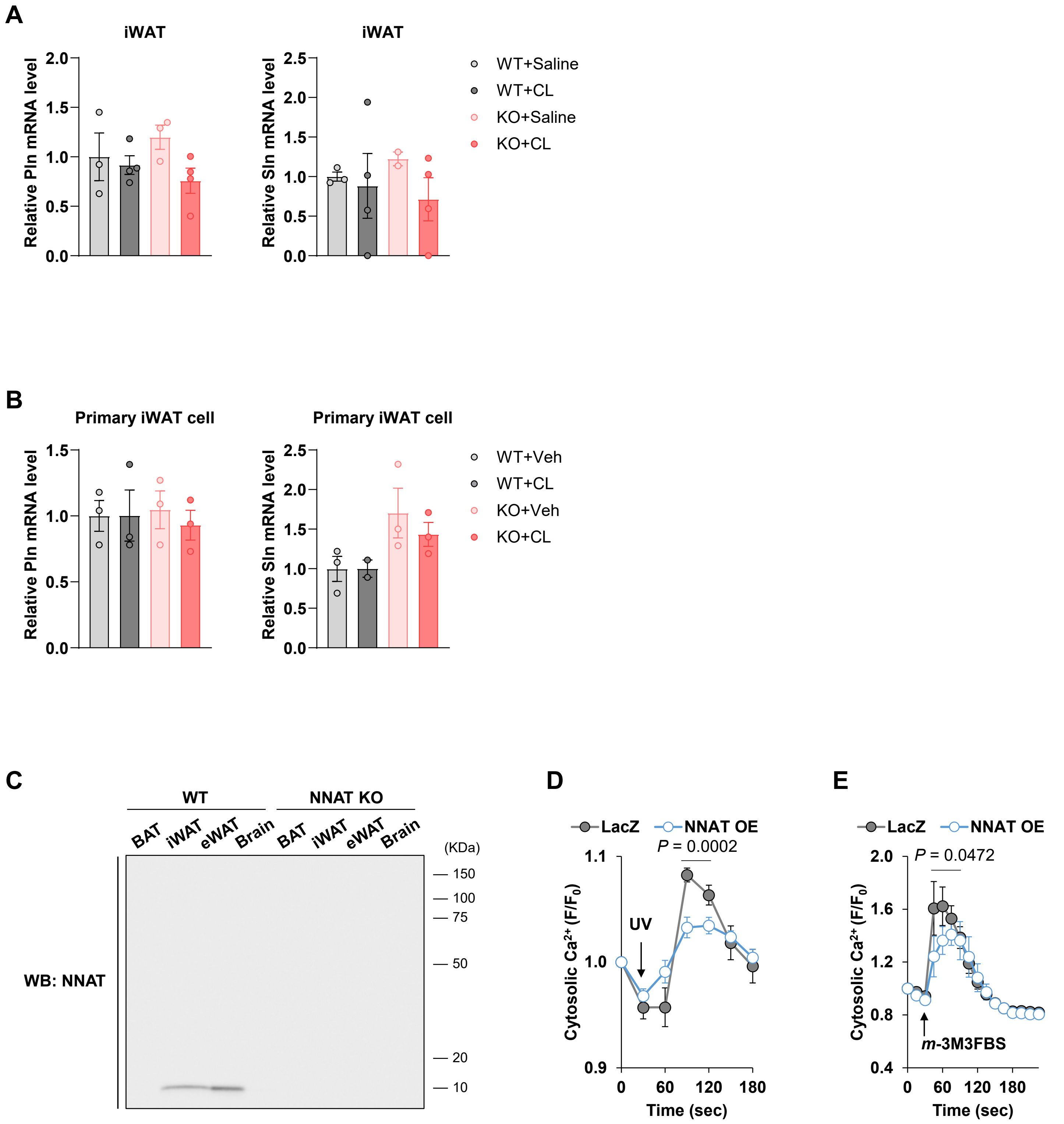

Supplementary Figure 4. Regulation of ER Ca^{2+} flux by NNAT

(A) Relative sarcolipin and phospholamban mRNA levels in iWAT from WT and KO mice treated with saline or 1 mg/kg CL316,243 for 3 days. n = 3 to 4 for each group. *P*-value is determined by two-tailed Student's t-test. No significant differences were noted.

(B) Relative sarcolipin and phospholamban mRNA levels in primary iWAT cells derived from WT and *Nnat* KO

mice and treated with vehicle or 10 μM CL316,243 for 2 days. n = 3 each. *P*-value is determined by two-tailed Student's t-test. No significant differences were seen.

(C) Full-sized immunoblot with the NNAT monoclonal antibody. Indicated tissues from WT and *Nnat* KO mice were collected and lysed for immunoblot assay. The whole blot was probed with the NNAT monoclonal antibody. A single band was observed at around 10 kDa in WT, and no band above 100 kDa was seen, such as the band detected in the crosslinked sample in Fig. 4C.

(D) HeLa cells ectopically expressing *LacZ* control or *Nnat* were loaded with Fluo-4 AM and caged IP3. Change in fluorescence was recorded after UV-induced uncaging of the IP3. *Nnat* OE showed decreased Ca^{2+} flux compared to control. *P*-value (between 90 and 120 seconds) is determined by two-way ANOVA followed by Fisher's LSD test.

(E) HeLa cells ectopically expressing *LacZ* control or *Nnat* were treated with 25 μM *m*-3M3FBS (Sigma) or vehicle and intracellular Ca^{2+} was measured. *P*-value (between 45 and 90 seconds) is determined by two-way ANOVA followed by Fisher's LSD test.

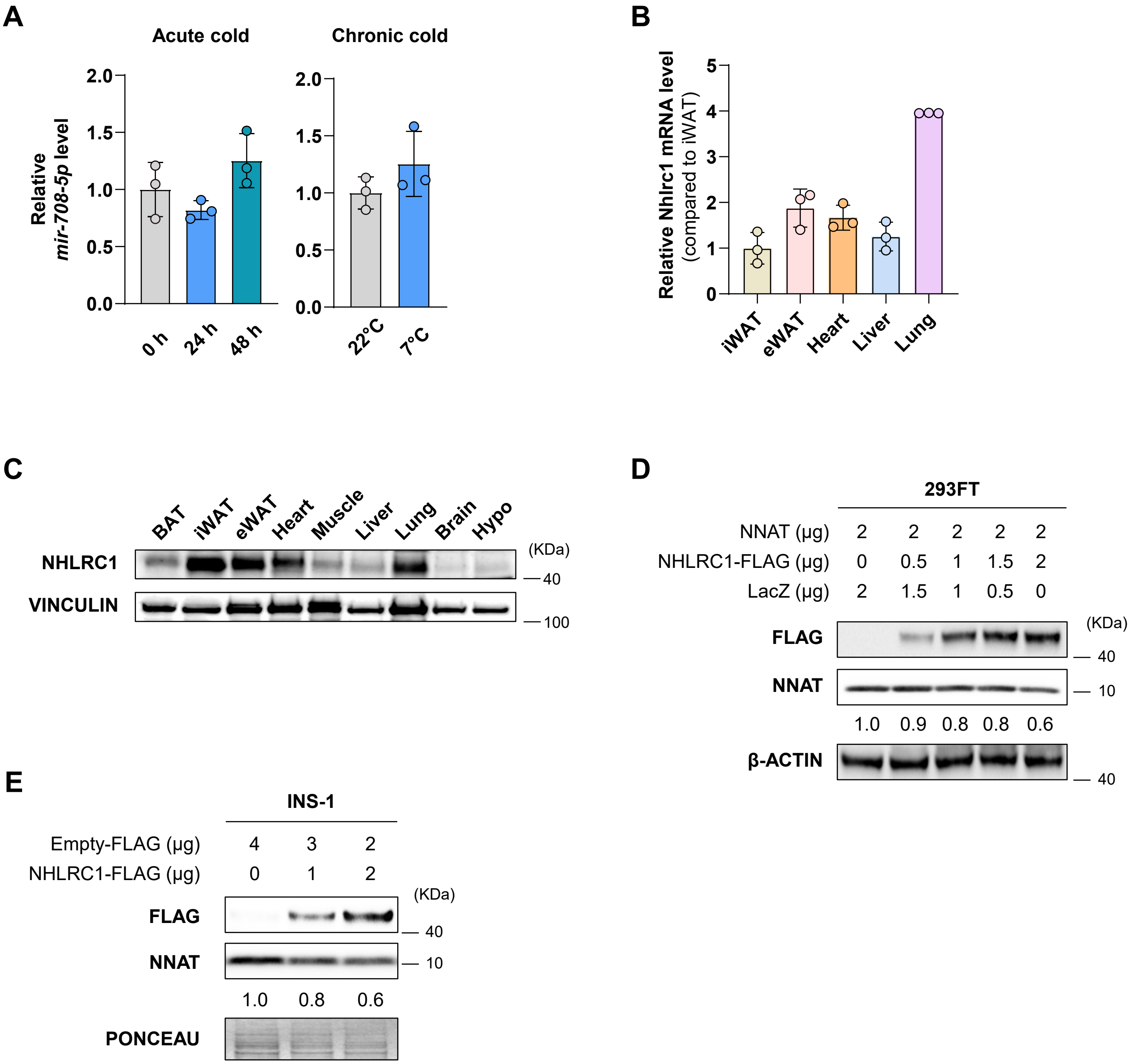

Supplementary Figure 5. Potential upstream regulators of NNAT in response to cold.

(A) Relative *mir-708-5p* expression in iWAT after acute cold and chronic cold exposure. *P*-value was determined by two-tailed Student's t-test. No significant changes were noted.

(B) Relative mRNA levels of *Nhlrc1* in the indicated tissues.

(C) NHLRC1 protein expression in various tissues.

(D) Increasing the NHLRC1 expression lowers the NNAT protein level in co-transfected 293FT cells.

(E) Ectopic expression of NHLRC1 reduces endogenous NNAT in INS-1 cells. For B and C, image quantitation was done using ImageJ and the relative amount of NNAT in each condition compared to zero NHLRC1 (lane 1) is shown.

Bar graphs are represented as mean ± SD.
